# Myofibroblast dedifferentiation proceeds via distinct transcriptomic and phenotypic transitions

**DOI:** 10.1172/jci.insight.144799

**Published:** 2021-03-22

**Authors:** Sean M. Fortier, Loka R. Penke, Dana King, Tho X. Pham, Giovanni Ligresti, Marc Peters-Golden

**Affiliations:** 1 Division of Pulmonary and Critical Care Medicine and; 2 BCRF Bioinformatics Core, University of Michigan, Ann Arbor, Michigan, USA.; 3 Department of Medicine, Boston University School of Medicine, Boston, Massachusetts, USA.

**Keywords:** Cell Biology, Pulmonology, Apoptosis, Fibrosis, Signal transduction

## Abstract

Myofibroblasts are the major cellular source of collagen, and their accumulation — via differentiation from fibroblasts and resistance to apoptosis — is a hallmark of tissue fibrosis. Clearance of myofibroblasts by dedifferentiation and restoration of apoptosis sensitivity has the potential to reverse fibrosis. Prostaglandin E_2_ (PGE_2_) and mitogens such as FGF2 have each been shown to dedifferentiate myofibroblasts, but — to our knowledge — the resultant cellular phenotypes have neither been comprehensively characterized or compared. Here, we show that PGE_2_ elicited dedifferentiation of human lung myofibroblasts via cAMP/PKA, while FGF2 utilized MEK/ERK. The 2 mediators yielded transitional cells with distinct transcriptomes, with FGF2 promoting but PGE_2_ inhibiting proliferation and survival. The gene expression pattern in fibroblasts isolated from the lungs of mice undergoing resolution of experimental fibrosis resembled that of myofibroblasts treated with PGE_2_ in vitro. We conclude that myofibroblast dedifferentiation can proceed via distinct programs exemplified by treatment with PGE_2_ and FGF2, with dedifferentiation occurring in vivo most closely resembling the former.

## Graphical abstract

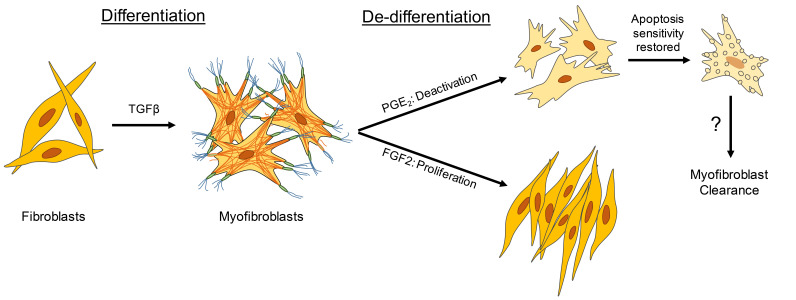

## Introduction

Fibrosis is the consequence of a disordered and pathologic host response to tissue injury that affects all organ systems and is associated with a high morbidity and mortality (1, 2). Idiopathic pulmonary fibrosis (IPF) is a severe and progressive lung disease characterized by parenchymal scarring that leads to architectural distortion, respiratory failure, and death (3). The ultimate effector cell of fibrotic disorders is the myofibroblast — a large stellate-shaped cell with numerous exocytotic vesicles, focal adhesions, and stress fibers composed of α–smooth muscle actin (αSMA). Lung myofibroblasts originate predominantly from resident fibroblasts whose further phenotypic differentiation is driven by profibrotic mediators, such as TGF-β. These cells promote fibrosis and tissue stiffness by virtue of their capacity to secrete abundant quantities of various extracellular matrix proteins such as collagens, elastin, and fibronectin and via their contractile function (4, 5). An additional important characteristic feature of myofibroblasts in IPF and other fibrotic disorders is their resistance to apoptosis, which contributes to their accumulation in involved tissues (6, 7). This accumulation of myofibroblasts contrasts with their eventual clearance during normal wound healing, thus allowing fibrosis to persist and progress (8).

Patients diagnosed with IPF usually come to clinical attention only after fibrosis is already established. Unfortunately, current therapies fail to reverse established fibrosis, in part because they do not eliminate the activated myofibroblasts that have accumulated. Although myofibroblasts were historically considered to be terminally differentiated cells, their capacity for dedifferentiation, defined as the loss of αSMA stress fibers, is now well recognized and has been implicated as being necessary for fibrosis resolution (9).

The 2 substances best established to promote dedifferentiation are the soluble mediators prostaglandin E_2_ (PGE_2_) (10–12) and mitogens such as FGF2 (13, 14). However, it is currently unclear how these 2 categories of molecules — which signal in entirely different ways — orchestrate the phenotypic transition of myofibroblasts. In the present study, we investigated this by performing mechanistic, RNA sequencing (RNA-seq), and functional studies on PGE_2_- and FGF2-treated human lung myofibroblasts examined in parallel. While treatment with PGE_2_ and FGF2 both downregulated hallmark fibrotic genes such as αSMA, collagen I, and fibronectin, they did so by generating distinct transitional cellular phenotypes, characterized by unique gene programs with differing capacities for proliferation and apoptosis. Finally, the gene signatures defined by RNA-seq of fibroblasts isolated from the lungs of young mice with resolving bleomycin-induced fibrosis were compared with those of myofibroblasts dedifferentiated in vitro and were found to more closely resemble those observed in cells treated with PGE_2_ than with FGF2.

## Results

### PGE_2_ utilizes the EP2/cAMP/PKA pathway to dedifferentiate myofibroblasts, whereas FGF2 requires MEK/ERK.

**Figure 1:**
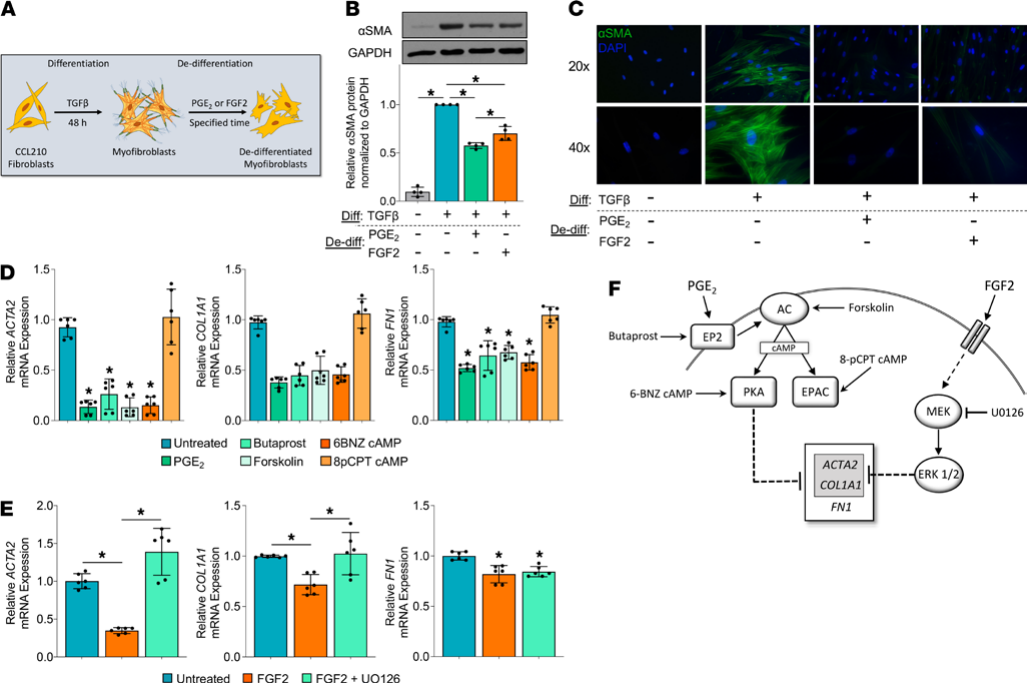
PGE_2_ and FGF2 dedifferentiate established myofibroblasts via distinct signaling pathways. (**A**) Experimental scheme depicting myofibroblast differentiation of CCL210 fibroblasts with TGF-β (2 ng/mL) for 48 hours, followed by dedifferentiation with PGE_2_ (1 μM) or FGF2 (50 ng/mL). (**B**) αSMA protein expression measured by Western blot analysis 5 days following treatment with PGE_2_ or FGF2 compared with untreated fibroblast and myofibroblast controls. The histogram depicts mean densitometry values. (**C**) αSMA stress fibers identified by immunofluorescence microscopy using anti-αSMA antibody and FITC-conjugated secondary antibody. Nuclei are stained with DAPI. (**D**) Relative *ACTA2*, *COL1A1*, and *FN1* expression by qPCR in myofibroblasts treated for 24 hours with PGE_2_ (1 μM), the EP2 agonist butaprost (500 nM), the adenylyl cyclase activator forskolin (500 nM), the PKA specific cAMP analog 6-BNZ cAMP (2 mM), or the Epac specific cAMP analog 8-pCPT cAMP (2 mM). (**E**) Relative *ACTA2*, *COL1A1*, and *FN1* expression by qPCR in myofibroblasts treated for 48 hours with FGF2 (50 ng/mL) with and without the MEK/ERK inhibitor UO126 (20 μM). (**F**) Schematic detailing PGE_2_ signaling cascade via the EP2 receptor and FGF2 signaling through FGF2R via MEK/ERK. PKA mediates the reduction in *ACTA2*, *COL1A1*, and *FN1* elicited by PGE_2_, while MEK/ERK mediates the reduction in *ACTA2* and *COL1A1* elicited by FGF2. Relative fold changes of indicated genes measured by qPCR are normalized to *GAPDH*. Data are presented as mean ± SEM. Data points in **B** represent individual replicate samples from 4 separate experiments. Data points in **D** and **E** represent paired replicate samples from 3 experiments. Lines indicate conditions being compared. *P < 0.05, compared with untreated myofibroblast, 1-way ANOVA. Diff, differentiation; De-diff, dedifferentiation; AC, adenylyl cyclase.

The abilities of PGE_2_ and FGF2 to elicit myofibroblast dedifferentiation have been studied individually but have not previously been directly compared. As depicted in the experimental scheme in Figure 1A, myofibroblasts were first established by treating normal adult human lung fibroblasts with TGF-β for 48 hours in serum-free medium, followed by subsequent addition of PGE_2_ or FGF2 to initiate dedifferentiation. We initially evaluated the effects of each treatment on the characteristic fibrosis-associated protein and myofibroblast marker αSMA. As expected, myofibroblasts expressed much higher levels of αSMA protein than did fibroblasts. Moreover, both PGE_2_ and FGF2 reduced the expression of αSMA protein at 5 days. Notably, PGE_2_ elicited a greater decline in αSMA (Figure 1B). In parallel with this global decline in αSMA protein, its assembly into stress fibers — an essential component of the myofibroblast contractile machinery — was also disrupted by treatment with PGE_2_ and FGF2 (Figure 1C). These results confirm that PGE_2_ and FGF2 each have the capacity to dedifferentiate myofibroblasts.

We confirmed that they elicited parallel reductions in αSMA mRNA (*ACTA2*), as well as the highly expressed myofibroblast genes collagen I (*COL1A1*) and fibronectin (*FN1*), when analyzed at 24 hours and 48 hours, respectively (Figure 1, D and E). These time points were selected based on observed differences in the dedifferentiation kinetics triggered by the 2 mediators in pilot experiments. It is notable that this shared outcome for both mediators occurs despite marked differences in their molecular identity, the characteristics of their cognate receptors, and second messenger signaling cascades. PGE_2_ is a prostaglandin lipid mediator known to interact with 4 E-prostanoid GPCRs (EP1–EP4), which signal through the second messengers cAMP or calcium (15). In contrast, FGF2 signals through a receptor tyrosine kinase affecting multiple downstream pathways, including PI3K, protein kinase B (AKT), and the MAPKs (16). We therefore investigated the signaling pathways involved in PGE_2_- and FGF2-mediated myofibroblast dedifferentiation. We have shown previously that PGE_2_ prevents TGF-β–induced fibroblast differentiation mainly by activation of the EP2 receptor, which stimulates adenylyl cyclase to increase production of intracellular cAMP and activate protein kinase A (PKA) (17–19). Accordingly, we examined the abilities of these same signaling pathway components of PGE_2_ to elicit myofibroblast dedifferentiation. Both butaprost, a PGE_2_ analog that specifically activates EP2, and forskolin, a direct activator of adenylyl cyclase, reduced levels of *ACTA2*, *COL1A1*, and *FN1* (Figure 1, D and E). cAMP can act via 2 effector proteins, PKA and the guanine nucleotide exchange protein activated by cAMP (Epac). A cAMP analog that selectively activates PKA (6-BNZ cAMP) was also capable of dedifferentiation, whereas one that selectively activates Epac (8-pCPT cAMP) was not. Next, we explored FGF2 signaling and found that its ability to reduce expression of αSMA and collagen I was overcome by inhibition of the MEK/ERK pathway (Figure 1E and Supplemental Figure 1A; supplemental material available online with this article; https://doi.org/10.1172/jci.insight.144799DS1) but not by inhibition of the MAPKs p38 or JNK, or by inhibition of AKT (Supplemental Figure 1B). The distinctiveness of the signaling pathways by which the 2 mediators exert their effects is underscored by the fact that the effects of PGE_2_ were not abolished by treatment with the ERK inhibitor U0126 (Supplemental Figure 1C). The modest reduction in *FN1* by FGF2 was not abolished by inhibition of MEK/ERK, p38, JNK, or AKT (Supplemental Figure 1D). The signaling mechanisms by which PGE_2_ and FGF2 promote myofibroblast dedifferentiation are summarized in Figure 1F.

### Transcriptome profiling of PGE_2_- and FGF2-treated myofibroblasts via RNA-seq.

**Figure 2:**
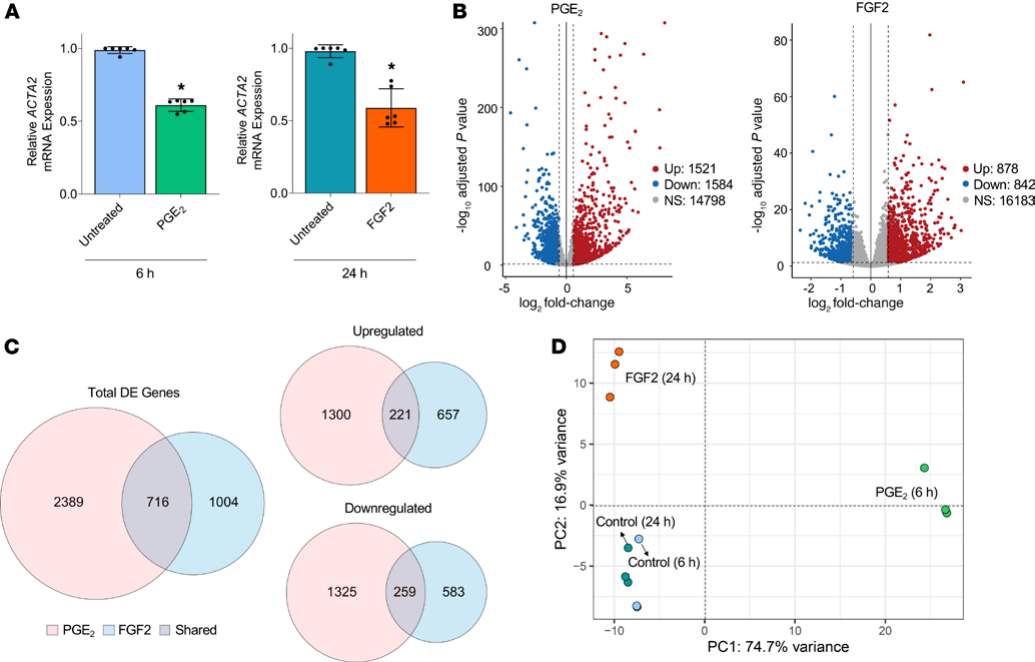
RNA-seq of established myofibroblasts treated with or without PGE_2_ or FGF2. (**A**) qPCR analysis of RNA samples submitted for RNA-seq demonstrating approximately 50% reduction in *ACTA2* expression in myofibroblasts treated with PGE_2_ (1 μM) or FGF2 (50 ng/mL) at 6 and 24 hours, respectively. Data points represent paired replicate samples from 3 experiments. Data are presented as mean ± SEM. *P < 0.05, paired 2-tailed *t* test. (**B**) Volcano plots representing differential gene expression by log_2_ fold change (*x* axis) and adjusted *P* value (*y* axis) of total RNA transcripts in PGE_2_- and FGF2-treated myofibroblasts compared with time-matched controls. (**C**) Venn diagrams depicting the number of genes differentially expressed as well as those specifically upregulated and downregulated exclusively by PGE_2_ (red), exclusively by FGF2 (blue), and by both mediators (gray). (**D**) Principal components analysis of the top 500 variably expressed genes in PGE_2_- and FGF2-treated myofibroblasts and untreated time-matched controls. Relative fold changes of indicated genes measured by qPCR are normalized to *GAPDH*. Each colored circle denotes 1 of 3 replicate samples.

Although we previously utilized microarray analysis to describe the transcriptomic effects of PGE_2_ in TGF-β–elicited myofibroblasts (12), that analysis lacked a comprehensive examination of noncoding RNAs (ncRNAs), as well as a comparison with the effects of FGF2. The fact that PGE_2_ and FGF2 led to myofibroblast dedifferentiation by exploiting different signaling pathways provided the impetus to undertake a more comprehensive examination that might reveal differences in the transitional cell phenotypes that they elicit. We interrogated this possibility using RNA-seq to characterize genome-wide transcriptomic changes in myofibroblasts treated with either PGE_2_ or FGF2. We sought to characterize early and comparable phases of the phenotypic transition promoted by PGE_2_ and FGF2. The time point arbitrarily chosen for analysis was that which yielded an approximately 50% reduction in *ACTA2*, determined to be 6 hours for PGE_2_ and 24 hours for FGF2 (Figure 2A). Each treatment condition was paired with a time-matched untreated myofibroblast control, and all analyses were performed in triplicate cultures. A total of 17,903 distinct RNA transcripts (excluding small RNAs) was detected. A high degree of concordance was observed among the triplicate samples from each treatment condition (Supplemental Figure 2). PGE_2_ differentially regulated 3105 genes, and FGF2 differentially regulated 1720 genes, as compared with their corresponding time-matched untreated controls. Of these, PGE_2_ upregulated 1521 genes and downregulated 1584, whereas FGF2 upregulated 878 and downregulated 842 (Figure 2B). PGE_2_ exhibited higher log-fold changes among top-regulated mRNAs than did FGF2, with only 1 gene — *DEPP1*, a regulator of autophagy (20) — shared among the top 25 genes up- or downregulated by both mediators (Supplemental Table 1). Despite their distinct actions, PGE_2_ and FGF2 mutually regulated 716 genes in common (Figure 2C). Principle components analysis showed similar clustering between 6 and 24 hours untreated controls but showed marked differences between myofibroblasts treated with PGE_2_ versus FGF2, as well as between each agent and the untreated controls (Figure 2D).

### Effects of PGE_2_ and FGF2 on myofibroblast signaling pathways and gene ontology.

We employed KEGG pathway enrichment analysis to characterize and compare the gene expression patterns of the transitional cellular phenotypes evoked by treatment of myofibroblasts with PGE_2_ and FGF2. Of the KEGG pathways included in our analysis, PGE_2_ and FGF2 each enriched 46 pathways, 17 of which were shared (Supplemental Table 2). From these, we curated a list of pathways lacking disease specificity, extrapulmonary organ selectivity, and functional redundancy (Table 1). Of these 22 remaining pathways, 14 were enriched by PGE_2_, 16 were enriched by FGF2, and 8 pathways were enriched by both.

Among the pathways uniquely enriched by PGE_2_ were MAPK and mTOR. Each has been shown to be involved in fibroblast biology in the context of pulmonary fibrosis (21, 22), with mTOR representing a potential target for antifibrotic therapy (23). In contrast, FGF2 predictably enriched cell cycle, apoptosis, and adhesion/anchoring pathways. Of particular interest were cellular functions enriched by both PGE_2_ and FGF2, given the possibility that a common pathway downstream of cAMP/PKA and MEK/ERK may participate in mediating the dedifferentiating effects of each molecule. Indeed, both PGE_2_ and FGF2 significantly impacted the fibrosis-associated pathways TGF-β, WNT, and PI3K-AKT. Notably, cAMP signaling was enriched by FGF2, as well as PGE_2_ (Table 1).

**Figure 3:**
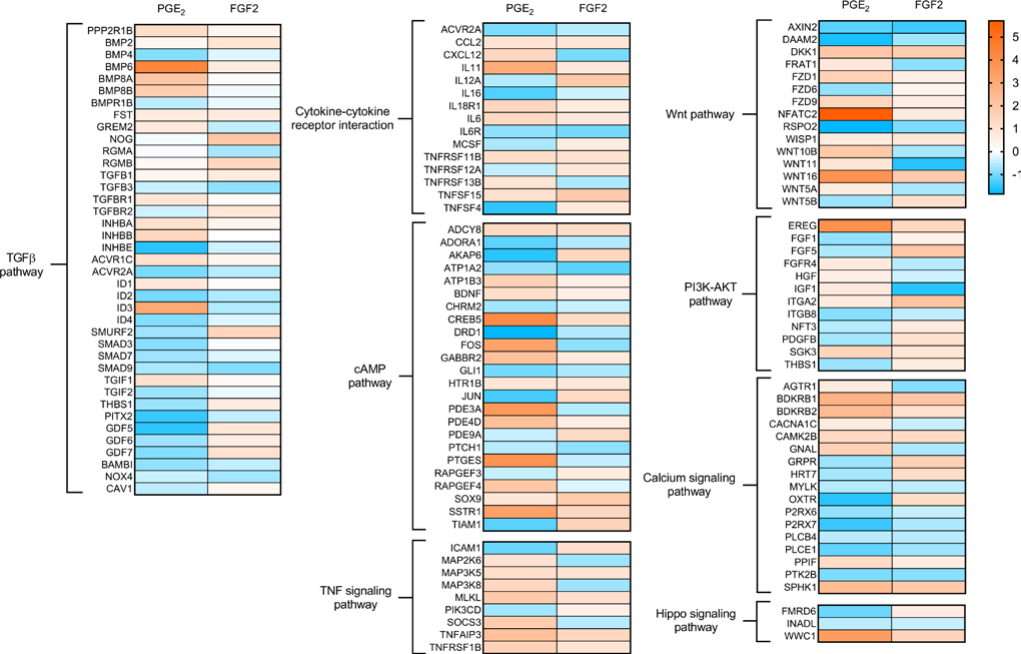
Regulation of genes from KEGG pathways enriched by both PGE_2_ and FGF2. Heatmap display of individual genes belonging to the specified pathways in myofibroblasts treated with PGE_2_ or FGF2 for 6 and 24 hours, respectively. Color scale depicts range of log_2_ fold changes in gene expression.

As TGF-β plays an integral role in the initiation, maintenance, and progression of pulmonary fibrosis (24–26), we specifically examined and compared the regulation of known TGF-β–associated genes. Broadly, PGE_2_ downregulated 21 TGF-β pathway genes, while FGF2 downregulated 10 (Figure 3). The canonical TGF-β effectors *SMAD3*, *SMAD7*, and *SMAD9* were downregulated by PGE_2_, while FGF2 downregulated *SMAD7* and *SMAD9* without significant regulation of *SMAD3*. Notably, the NADPH oxidase gene *NOX4* — a central mediator of myofibroblast differentiation, ECM protein production, and contractility (27) — was downregulated by both effector molecules.

We next compared differentially expressed genes belonging to the remaining mutually enriched pathways (Figure 3). PGE_2_ and FGF2 upregulated cytokines such as *IL6*, *IL11*, and *CCL2*, while downregulating *IL16* and *IL6R*. Interestingly, *SOCS3* — which opposes IL6 signaling by inhibiting JAK-STAT — was upregulated by PGE_2_ and downregulated by FGF2. Though many genes belonging to the cAMP signaling pathway were regulated in parallel by each treatment, the hallmark PKA-induced early gene *FOS* was repressed by FGF2, while *JUN*, whose protein product commonly binds FOS to form the AP-1 complex, was repressed by PGE_2_. It is notable that both of these subunits of the archetypal AP-1 heterodimer — which has been shown to promote fibrosis (28, 29) — were reduced by either PGE_2_ or FGF2. The WNT-associated genes *NFATC2*, *FZD1*, *FZD9*, and *WISP1* were upregulated by both treatments, whereas *WNT5A*, *WNT5B*, *WNT10B*, and *WNT11* were induced by PGE_2_ and repressed by FGF2. The growth factors *FGF1*, *FGF5*, and *PDGFB* were all downregulated by PGE_2_ and upregulated by FGF2, whereas *HGF*, shown to be antifibrotic (30) and deficient in IPF fibroblasts (31), was upregulated by PGE_2_ but downregulated by FGF2. These results highlight many differences in gene regulation between PGE_2_ and FGF2, even within mutually enriched pathways.

**Figure 4:**
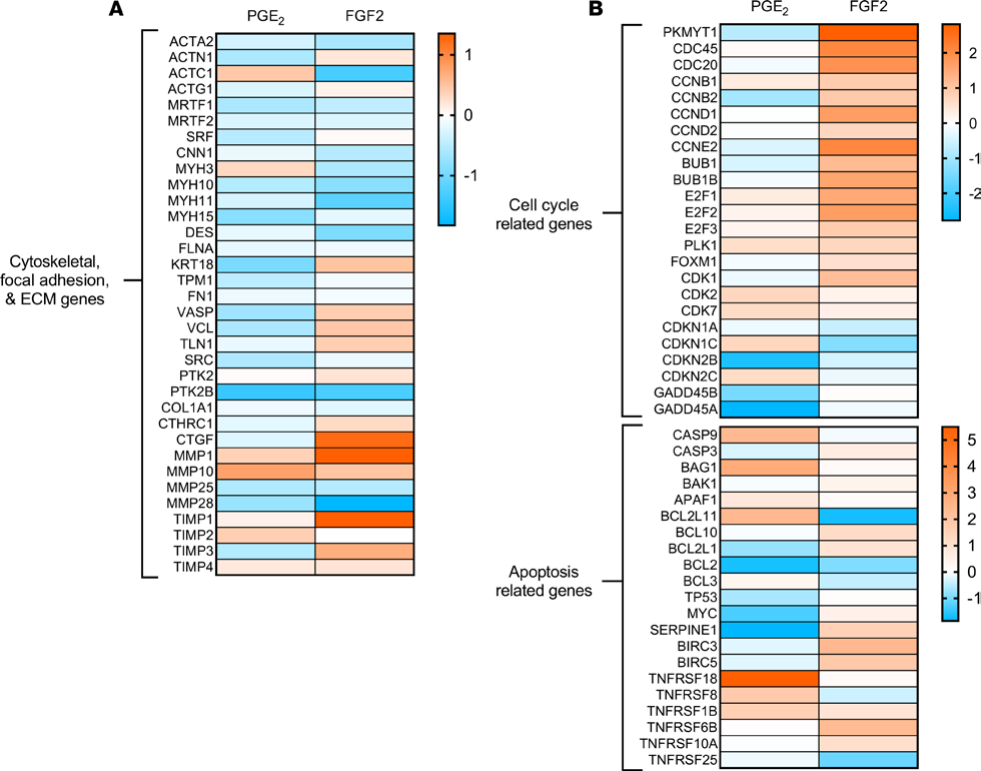
Gene ontology characteristics of myofibroblasts following treatment with PGE_2_ or FGF2. (**A** and **B**) Heatmap display of cytoskeletal, ECM-related, and focal adhesion (**A**) and cell cycle and apoptosis genes (**B**) in PGE_2_ - and FGF2-treated myofibroblasts compared with 6- and 24-hour time-matched controls. Color scale depicts range of log_2_ fold changes in gene expression. ECM, extracellular matrix.

As discussed above, tissue contraction, ECM protein secretion, and apoptosis resistance are cardinal features of myofibroblasts. We therefore specifically interrogated the gene ontologies associated with these biologic processes. First, we found that PGE_2_, and to a lesser extent FGF2, downregulated a number of important cytoskeletal, focal adhesion, and ECM-related genes (Figure 4A), including *ACTA2* and *ACTN1*. Both treatments reduced the expression of *MRTF1*, a transcription factor crucial for TGF-β–induced expression of αSMA in lung myofibroblasts (17). Neither treatment resulted in a significant change in *FN1* or *COL1A1* expression at the early time points at which sequencing analysis was performed, although each of these transcripts was reduced at later time points (Figure 1). Notably, PGE_2_ suppressed the focal adhesion mediators *VASP*, *VLC*, *TLN1*, and *CTHRC1*, while FGF2 increased the expression of each. Though focal adhesion kinase (*PTK2*) was not significantly modulated by either treatment, the related gene *PTK2B*, which has similar biologic endpoints as *PTK2* (32), was markedly downregulated by both. Modulation of the extracellular matrix metalloproteases (MMPs) and their tissue inhibitors of metalloproteinase (TIMPs) was heterogeneous between treatments other than the mutual suppression of *MMP25* and *MMP28*. Finally, the pleiotropic growth factor *CTGF* was inversely regulated — decreased by PGE_2_ and induced by FGF2.

Cellular differentiation and proliferation have long been considered to represent opposing or even mutually exclusive cellular programs (33). Indeed, it has been suggested that such a mechanism is integral to the ability of mitogens such as FGF2 to promote dedifferentiation (9). Predictably, myofibroblasts treated with FGF2 upregulated multiple cell cycle genes, including cyclins (*CCNB1*, *CCNB2*, *CCND1*, *CCND2*, and *CCNE2*), cyclin-dependent kinases (*CDK1*, *CDK2*, and *CDK7*), and *FOXM1* — a critical transcription factor in fibroblast proliferation (34) (Figure 4B). By contrast, PGE_2_, which is well known to inhibit mesenchymal cell proliferation (33, 35, 36), exerted minimal effects on the expression of these genes and upregulated the cyclin-dependent kinase inhibitors *CDKN1C* and *CDKN2C*. Consistent with its proproliferative effects, FGF2 increased prosurvival genes such as *BIRC5* and *MYC*, whereas PGE_2_ increased the proapoptotic genes *APAF1* and *CASP9*. Finally, the profibrotic protein *SERPINE1*, well known to be overexpressed in IPF fibroblasts and reported to contribute to apoptosis resistance in myofibroblasts (37), was downregulated by PGE_2_ but upregulated by FGF2 (Figure 4B). These results indicate that PGE_2_ and FGF2 have largely opposing effects on genes involved in proliferation and apoptosis.

### PGE_2_ and FGF2 modulate the expression of fibrosis-associated long noncoding RNAs and microRNAs in myofibroblasts.

**Figure 5:**
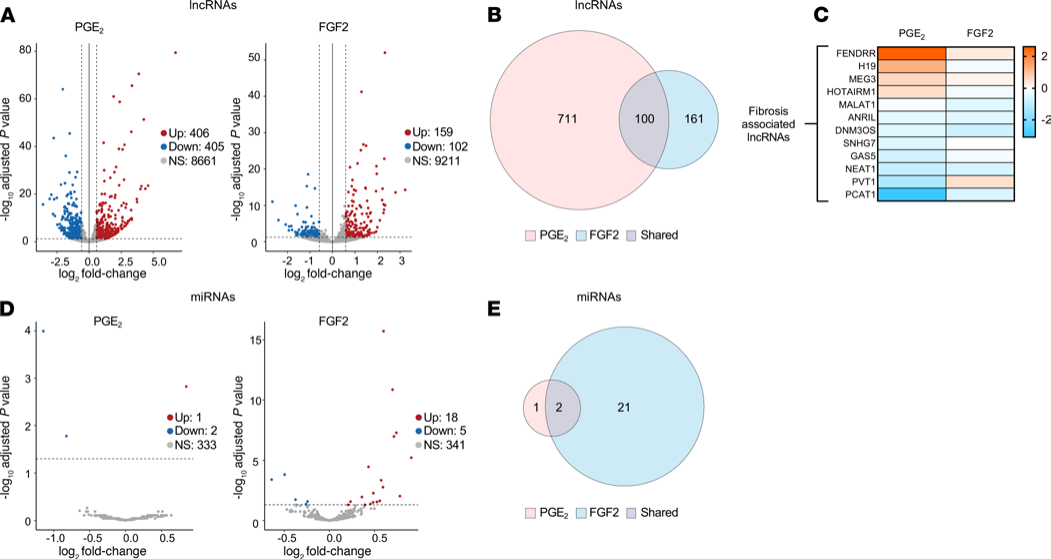
PGE_2_ and FGF2 modulate the expression of fibrosis-associated long noncoding RNAs and miRNAs in myofibroblasts. (**A** and **D**) Volcano plots representing differential lncRNA expression (**A**) miRNA expression (**D**) by log_2_ fold change (*x* axis) and adjusted *P* value (*y* axis) of total RNA transcripts in PGE_2_- and FGF2-treated myofibroblasts compared with time-matched controls. Threshold for lncRNAs set by log_2_ fold change –0.5 to 0.5 and adjusted *P* < 0.05. Threshold for miRNAs set by adjusted *P* < 0.05 only. (**B** and **E**) Venn diagrams depicting the number of differentially expressed lncRNAs (**B**) and miRNAs (**E**) exclusively by PGE_2_ (red), exclusively by FGF2 (blue), and by both mediators (gray). (**C**) Heatmap display of fibrosis-associated lncRNAs differentially regulated by PGE_2_ and/or FGF2. Color scale depicts range of log_2_ fold changes in gene expression.

ncRNAs play significant roles in posttranscriptional regulation, as well as the determination and maintenance of cell phenotypes (38). Among these, a number of miRNAs exert pro- or antifibrotic effects by inhibiting translation and promoting RNA degradation (39, 40). Similarly, many long noncoding RNAs (lncRNAs) influence fibrosis through various mechanisms including miRNA “sponging” — preventing miRNA-mRNA interactions by competitive binding (41). Of the more than 8800 total lncRNAs identified, PGE_2_ and FGF2 modulated 811 and 261, respectively, with 100 shared among them (Figure 5, A and B). The top 25 regulated lncRNAs are listed in Supplemental Table 3. We curated a list of fibrosis-associated lncRNAs regulated by at least 1 of our treatments (Figure 5C). Both treatments downregulated *NEAT1* and *DNM3OS*, while PGE_2_ reduced the profibrotic *PCAT1* and *PVT1*, and FGF2 reduced *MALAT1*. The abundant lncRNAs *NEAT1* and *MALAT1* have been shown to contribute to organ fibrosis and to sponge numerous miRNAs (42, 43), while *DNM3OS* has been reported to promote the expression of the profibrotic miR–214-3p and miR–199a-3p/5p in human lung fibroblasts (44). PGE_2_ also increased the antifibrotic lncRNA *FENDRR*, which sponges miR–214-3p and is known to be reduced in IPF fibroblasts (45).

Though over 350 miRNAs were detected under each treatment condition, PGE_2_ only significantly modulated 3 miRNAs while FGF2 regulated 23 (Figure 5, D and E, Supplemental Table 4). Both PGE_2_ and FGF2 increased expression of miR-543, which has been shown to inhibit TGF-β activation and gene expression in rat cardiac fibroblasts (46). miR–335-3p, an antiproliferative and proapoptotic miRNA with antifibrotic properties in gingival fibroblasts (47, 48), was upregulated by PGE_2_ and repressed by FGF2. The top PGE_2_-upregulated miRNA, miR–129-5p, exhibits antifibrotic actions in dermal fibroblasts by targeting *COL1A1* (49). Furthermore, FGF2 upregulated the antifibrotic miR–29b-3p/5p, miR–152-5p, and let-7a-3p (5, 50, 51), while downregulating the profibrotic miR–145-3p (52). Other profibrotic- and antifibrotic-associated miRNAs — including miR-21 (53) and miR–27a-3p/miR–27b-3p (54) — were detected in high abundance in our analysis but were not differentially regulated by either treatment (Supplemental Table 5). As discussed above, miRNA activity can be regulated through RNA sponging without changes in miRNA expression (38, 55). Indeed, multiple differentially expressed lncRNAs in our analysis have been shown to affect the function of a number of fibrosis-associated miRNAs that were not, themselves, differentially regulated in our study (Table 2). These data indicate a possible role for ncRNAs in PGE_2_- and FGF2-mediated dedifferentiation of myofibroblasts.

### Myofibroblasts treated with PGE_2_ and FGF2 separately or in combination produce distinct cellular morphologies and fibrosis-associated gene expression patterns.

**Figure 6:**
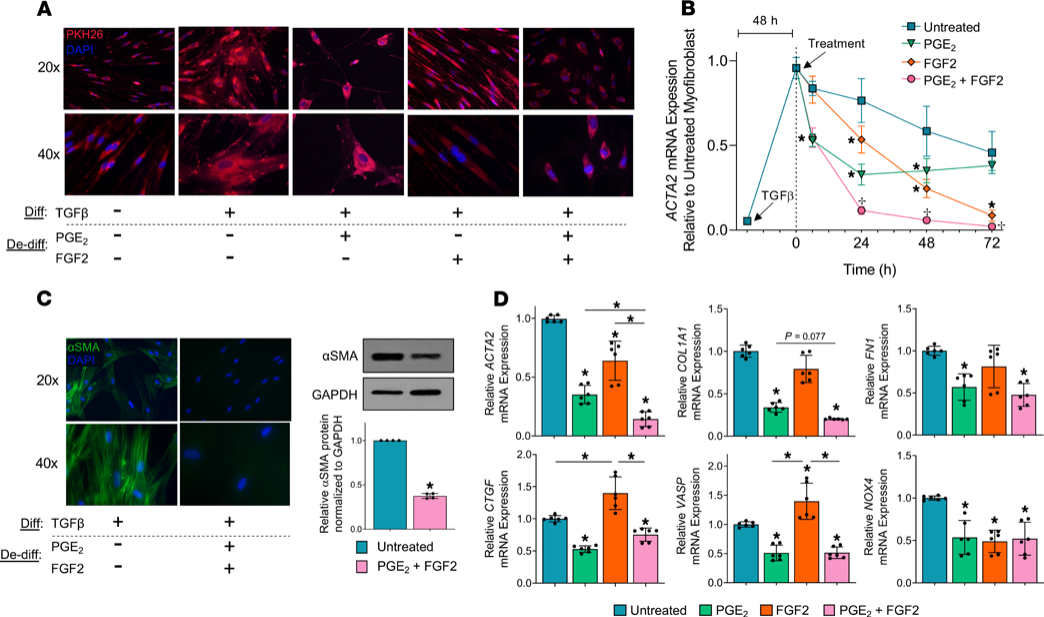
Myofibroblasts treated with PGE_2_ and FGF2 separately or in combination produce distinct cellular morphologies and fibrosis-associated gene expression patterns. CCL210 fibroblasts were differentiated to myofibroblasts with TGF-β (2 ng/mL) and treated with PGE_2_ (1 μM), FGF2 (50 ng/mL), or both. (**A**) Cells were stained with the membrane dye PKH26 (2 μM) and examined by fluorescence microscopy 5 days after treatment. (**B**) Kinetics of *ACTA2* in untreated, PGE_2_-, FGF2-, and PGE_2_ + FGF2–treated myofibroblasts. Fibroblasts were treated with TGF-β for 48 hours, followed by treatment and harvesting for mRNA at the indicated time points. (**C**) Immunofluorescence microscopy and representative Western blot for αSMA in untreated and PGE_2_ + FGF2–treated myofibroblasts evaluated at 5 days. The histogram depicts mean densitometry values. (**D**) qPCR analysis of the fibrosis-associated genes *ACTA2*, *COL1A1*, *FN1*, *CTGF*, *VASP*, and *NOX4* after 24 hours of PGE_2_ ± FGF2 compared with untreated myofibroblast control. Relative fold changes of indicated genes measured by qPCR are normalized to *GAPDH*. Data are presented as mean ± SEM; data points represent replicate samples from 3 experiments. Lines indicate conditions being compared. *Statistical significance compared with untreated myofibroblast; ^+^Statistical significance compared with untreated, PGE_2_-, and FGF2-treated myofibroblasts. *P < 0.05 and ^+^*P* < 0.05. Performed 2-way ANOVA for **B**, paired 2-tailed *t* test for **C**, and 1-way ANOVA for **D**. Diff, differentiation; De-diff, dedifferentiation.

To complement their distinct transcriptomic effects, we sought to examine the influence of PGE_2_ and FGF2 on myofibroblast morphology. Furthermore, we wished to determine the net effect of combining these 2 effector molecules — a closer approximation of the in vivo conditions in which they would be expected to coexist — on cell morphology, loss of stress fibers, and the expression of various fibrosis-associated genes. Undifferentiated fibroblasts stained with the membrane dye PKH26 appeared spindle shaped and elongated, in stark contrast to the larger, cuboidal, and stellate-shaped myofibroblasts (Figure 6A). Compared with untreated myofibroblasts, those subsequently exposed to PGE_2_ appeared smaller and thinner, and they displayed fewer cytoplasmic projections. FGF2-treated myofibroblasts lost their stellate shape, appearing thin and elongated, similar to native fibroblasts. The combination of PGE_2_ and FGF2 produced a small, more rounded cell, distinct from the transitional cell morphology produced by either treatment alone. We assessed the kinetics of *ACTA2* in response to each treatment, as well as their combination (Figure 6B). Combined treatment displayed an effect that was statistically greater than that with either treatment alone at 24, 48, and 72 hours. Interestingly, though PGE_2_ treatment resulted in a more rapid reduction in *ACTA2* through 24 hours, its effects plateaued at 48 hours, whereas FGF2 progressively reduced *ACTA2* through 72 hours. Combined treatment also reduced αSMA protein and its organization into stress fibers (Figure 6C) to an extent apparently greater than did either treatment alone (Figure 1B). We next performed quantitative PCR (qPCR) on selected fibrotic genes to further evaluate the effect of combined treatment on each (Figure 6D). As displayed in Figure 6B, combined treatment with PGE_2_ and FGF2 reduced *ACTA2* at 24 hours to a greater extent than either treatment alone. There was a trend toward an additive reduction in *COL1A1*. *FN1* reduction in response to PGE_2_ and FGF2 appeared comparable in magnitude to PGE_2_ alone. Though the profibrotic growth factor *CTGF* and focal adhesion regulator *VASP* were each downregulated by PGE_2_ and upregulated by FGF2, combination treatment resulted in reduced expression of each transcript. Expression of *NOX4* was reduced in PGE_2_- and FGF2-treated myofibroblasts, though combination treatment did not yield any further reduction.

### PGE_2_ and FGF2 have opposite effects on myofibroblast proliferation and apoptosis.

**Figure 7:**
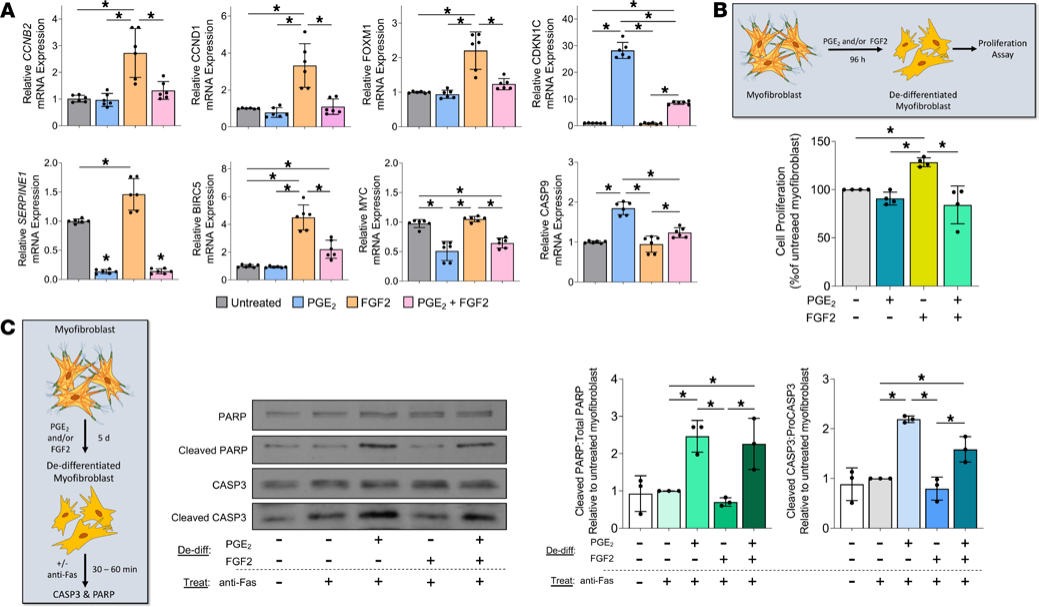
PGE_2_ and FGF2 have opposite effects on myofibroblast proliferation and apoptosis. (**A**) CCL210 myofibroblasts were treated with PGE_2_, FGF2, or PGE_2_ + FGF2 for 24–72 hours. qPCR analysis of the proliferation gene *FOXM1* was performed at 48 hours, while *CCNB2*, *CCND1*, and *CDKN1C* were assessed at 72 hours (top panel). qPCR analysis of the antiapoptotic gene *SERPINE1* was performed at 24 hours, while *BIRC5* and *MYC* were assessed at 48 hours; the proapoptotic gene *CASP9* was assessed at 72 hours. (**B**) Proliferation was assessed 96 hours following treatment with PGE_2_ and/or FGF2 by CyQUANT Cell Proliferation Assay. (**C**) Apoptosis sensitivity was assessed by measuring total and cleaved CASP3 and PARP by Western blot analysis in myofibroblasts 5 days following addition of PGE_2_ and/or FGF2, followed by treatment with the death receptor ligand anti-Fas. CASP3 was measured 30 minutes and PARP 1 hour following anti-Fas treatment. Densitometry represents ratio of cleaved products to total protein. Relative fold changes of indicated genes measured by qPCR are normalized to *GAPDH*. Data are presented as mean ± SEM. Data points represent replicate samples from 3 (**A** and **C**) or 4 (**B**) experiments. Lines indicate conditions being compared. *P < 0.05, compared with untreated myofibroblast; 1-way ANOVA. De-diff, dedifferentiation.

Our transcriptomic analysis predicted that treatment of myofibroblasts with PGE_2_ would inhibit their capacity for proliferation and reestablish apoptosis sensitivity, while treatment with FGF2 would promote proliferation and maintain apoptosis resistance. We tested these predictions by directly assaying proliferation capacity and apoptosis sensitivity in myofibroblasts treated with PGE_2_ and/or FGF2 as compared with untreated controls. First, we assessed the expression of cell cycle genes (Figure 7A, top panel) and apoptosis genes (Figure 7A, bottom panel) in myofibroblasts exposed to each treatment alone and in combination. *CCNB2*, *CCND1*, and *FOXM1* were upregulated by FGF2, consistent with our RNA-seq data. Importantly, though PGE_2_ did not significantly modulate these genes, it negated their upregulation by FGF2. Conversely, the expression of the cyclin-dependent kinase inhibitor *CKDN1C* was greatly and uniquely increased by PGE_2_, an increase not fully abrogated by addition of FGF2. The antiapoptotic genes *BIRC5* and *SERPINE1* were each uniquely upregulated by FGF2, but such increases were partially or completely abrogated by the addition of PGE_2_, while *MYC* was downregulated by PGE_2_ in the presence or absence of FGF2. The proapoptotic gene *CASP9*, which leads to activation of CASP3 (56), was upregulated by PGE_2_ and downregulated by FGF2, and it remained slightly upregulated with combined treatment. Taken together, these data demonstrate that PGE_2_ and FGF2 have opposing effects on myofibroblast genes associated with proliferation and apoptosis.

We next determined if these transcriptomic differences were associated with functional differences in myofibroblast proliferation and apoptosis. Consistent with the effect of each treatment on cell cycle genes, FGF2 increased the proliferation of established myofibroblasts, whereas PGE_2_ had no effect relative to untreated myofibroblasts but abolished the proliferative effects of FGF2 (Figure 7B). To assess the apoptosis sensitivity of myofibroblasts undergoing dedifferentiation, we treated cells with PGE_2_ and/or FGF2 for 5 days prior to treatment with an anti-Fas activating antibody; apoptosis was assessed by Western blot detection of cleaved/total CASP3 and PARP within cell lysates (Figure 7C). As expected, the levels of cleaved CASP3 and PARP did not increase in response to Fas activation of untreated myofibroblasts, highlighting their intrinsic resistance to apoptosis. In contrast, myofibroblasts dedifferentiated with PGE_2_ displayed significantly higher levels of cleaved CASP3 and PARP after exposure to anti-Fas activating antibody relative to untreated myofibroblasts. Such an effect was not seen in myofibroblasts dedifferentiated with FGF2, but it was preserved in myofibroblasts dedifferentiated with both agents together. To assess whether restoration of apoptosis sensitivity in myofibroblasts elicited by PGE_2_ depends on prior dedifferentiation, we performed immunofluorescence microscopy for annexin V and αSMA in PGE_2_-treated myofibroblasts and untreated myofibroblast controls subsequently exposed to anti-Fas for 4 hours (Supplemental Figure 3). Notably, we found a small proportion of PGE_2_-treated myofibroblasts that retained their stress fibers, suggesting underlying fibroblast heterogeneity. PGE_2_-treated myofibroblasts undergoing apoptosis — defined by annexin V staining — were almost exclusively limited to those lacking αSMA stress fibers. Untreated myofibroblasts did not exhibit annexin V staining, further confirming their resistance to apoptosis.

### Lung fibroblasts from an in vivo model of fibrosis resolution exhibit similar gene signatures as those determined in myofibroblasts dedifferentiated in vitro.

**Figure 8:**
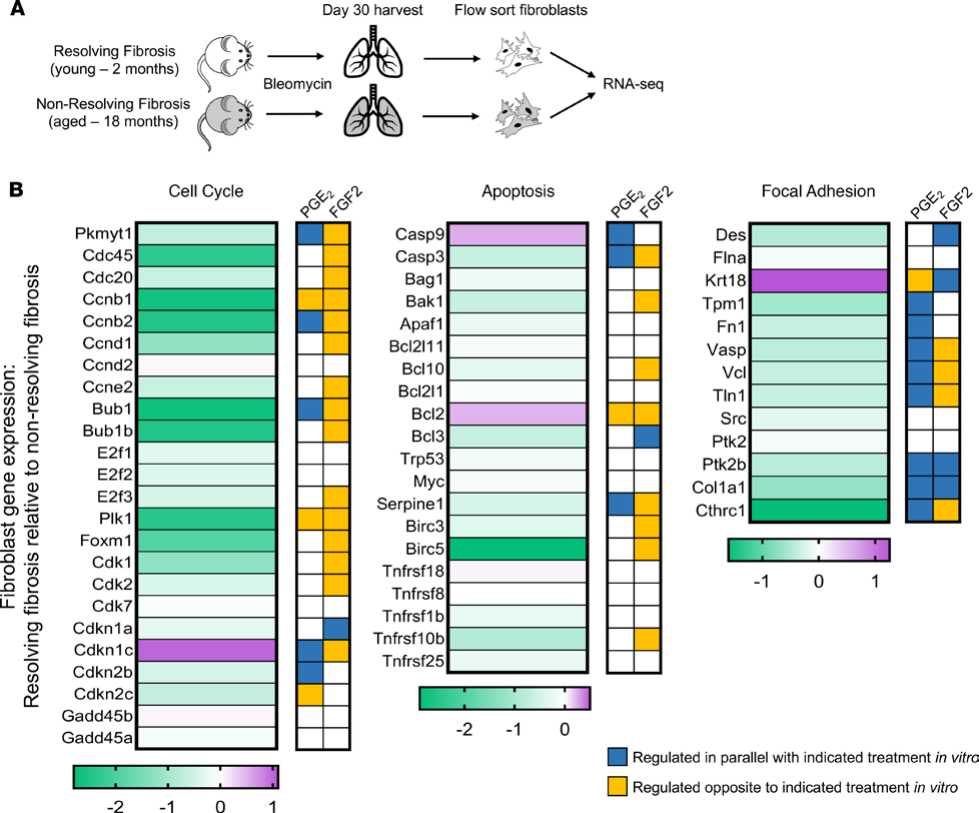
Lung fibroblasts from an in vivo model of fibrosis resolution exhibit similar gene signatures as those determined in myofibroblasts dedifferentiated in vitro. (**A**) Experimental scheme for bleomycin-induced pulmonary fibrosis in Col1α1-GFP^+^ mice with resolving fibrosis (young) and nonresolving fibrosis (aged); mice were sacrificed on day 30, and fibroblasts were flow sorted from lungs and submitted for RNA-seq. (**B**) Heatmap display of gene expression in mice with resolving fibrosis (compared with the expression in mice with nonresolving fibrosis). Color scale depicts range of log_2_ fold changes in gene expression. *Tnfrsf10b* is the mouse homolog of human *TNFRSF10A*. Gene expression patterns regulated in parallel (blue) or opposite (yellow) to those exhibited with in vitro treatments of human myofibroblasts are indicated in color-filled boxes to the right of the heatmaps.

The RNA-seq data presented thus far were derived from in vitro treatment of myofibroblasts with individual mediators. To evaluate the relevance of these findings in a less reductionist and in vivo model of fibrosis resolution, we compared the transcriptome of freshly isolated lung fibroblasts from young mice with that of lung fibroblasts isolated from aged mice following bleomycin challenge. It has been established that, in contrast to aged mice, young mice subjected to bleomycin-induced pulmonary fibrosis can undergo spontaneous resolution — in which fibroblasts reduce collagen secretion and lose their αSMA stress fibers (57–59). To characterize their global transcriptome, RNA-seq was performed on FAC-sorted fibroblasts isolated from the lungs of young mice (2 months) and aged mice (18 months) during the early phase of fibrosis resolution (30 days after bleomycin) (Figure 8A). A comprehensive analysis of these data is described in a separate manuscript currently in preparation; herein, we present limited data from this dataset relevant to cell cycle, apoptosis, and focal adhesion genes found to be significantly modulated by either PGE_2_ or FGF2 treatment of myofibroblasts in vitro (Figure 4, A and B). Gene expression changes in fibroblasts isolated from young lungs undergoing fibrosis resolution were expressed relative to those of fibroblasts isolated from aged mouse lungs with nonresolving fibrosis (Figure 8B).

Lung fibroblasts sorted from mouse lungs undergoing fibrosis resolution expressed lower levels of proliferation genes, including *Ccnb1*, *Ccnb2*, *Ccnd1*, *Ccne1*, *Cdk1*, and *Foxm1*. Conversely, the antiproliferative *Cdkn1c* was upregulated. Five of the eighteen significantly modulated cell cycle genes in fibroblasts from mice with resolving fibrosis were regulated in parallel to PGE_2_-treated human myofibroblasts, while just 1 gene was regulated in parallel to FGF2-treated myofibroblasts. Notably, 15 of these 18 cell cycle genes were regulated oppositely to that of FGF2-treated myofibroblasts in vitro, whereas only 3 genes were regulated opposite to PGE_2_-treated myofibroblasts. The proapoptotic gene *Casp9* was upregulated in fibroblasts from mice with resolving fibrosis, and the prosurvival genes *Birc5*, *Birc3*, and *Serpine1* were all downregulated. Of the 10 apoptosis-associated genes significantly modulated in young lung fibroblasts, 3 were regulated in parallel with PGE_2_-treated versus 1 in FGF2-treated myofibroblasts. Interestingly, the apoptosis-associated genes *Myc*, *Bcl2l1*, *Bcl2l11*, and *Tnfrsf8* — which were regulated oppositely by PGE_2_ and FGF2 in vitro — showed no significant differences between fibroblasts isolated from mice with resolving and nonresolving fibrosis, suggesting an in vivo level of expression reflecting the arithmetic sum of changes elicited individually by PGE_2_ and FGF2 in vitro. Eight of 10 significantly modulated focal adhesion genes were also downregulated in parallel with the PGE_2_-treated myofibroblasts. Among these were *Vasp* — a phosphoprotein involved in actin assembly and SRF activity (60) — and *Cthrc1*, which is a marker of tissue fibrosis (61). In contrast, FGF2-treated myofibroblasts showed opposite regulation of *Vasp*, *Vcl*, *Tln*, and *Cthrc1*. *Col1a1* and *PTK2B* were the only genes assessed among these pathways that were concordantly regulated by PGE_2_ and FGF2 treatment of myofibroblasts in vitro as well as in fibroblasts from mice during fibrosis resolution.

## Discussion

Myofibroblasts are critical effector cells that orchestrate, maintain, and propagate lung scarring in IPF and other fibrotic disorders (62). Whereas inhibiting the fibroblast to myofibroblast transition has the potential to prevent fibrogenesis in response to a recognized injury and to arrest progression of an established fibrotic process, it is unlikely to be sufficient to promote its resolution. By contrast, promoting myofibroblast clearance has the potential to do so. Since elimination of myofibroblasts in fibrotic diseases is limited by their resistance to apoptosis, dedifferentiation provides a potential route to restore apoptosis sensitivity and, thus, facilitate myofibroblast removal. Indeed, myofibroblast dedifferentiation has been reported in response to a variety of effector molecules, including mitogens such as serum and FGFs (13, 14), lipid mediators PGE_2_ and PGI_2_ (10, 63), statins (64), siRNA knockdown of the transcription factors MyoD (33) and FOXM1 (34), and the bromodomain inhibitor JQ1 (65). Whether dedifferentiation with these diverse classes of effector molecules yields common or divergent transitional cell phenotypes has not previously been explored. Here, we compared the operative signaling mechanisms, as well as the transcriptomic, morphologic, and functional characteristics, of myofibroblasts treated with the endogenous soluble mediators PGE_2_ and FGF2.

Our results confirm that PGE_2_ and FGF2 are both capable of dedifferentiating human lung myofibroblasts by downregulating αSMA and eradicating stress fibers (Figure 1, B and C). However, they accomplish this via distinct signaling pathways and with different kinetics (Figure 1, B, D, and E; and Figure 6B). Globally, PGE_2_ modulated nearly double the number of protein-coding genes and nearly triple the number of ncRNAs, and it induced greater log-fold changes among top-regulated genes than did FGF2 (Figure 2B, Figure 5A, and Supplemental Table 1). Moreover, each mediator produced a unique transitional cell phenotype characterized by distinctive gene programs, morphology, proliferative capacities, and sensitivity to apoptosis (Figures 3–7).

It is now recognized that myofibroblasts have multiple potential fates (8, 66–68). While myofibroblast senescence favors their persistence and, thus, progression of fibrotic disease (9), we demonstrate herein that deactivation and proliferation represent 2 distinct paths by which myofibroblasts can dedifferentiate. As in prior studies establishing that PGE_2_ inhibits many critical functions of fibroblasts — including proliferation, differentiation, contractility, and focal adhesion (17, 19, 69) — we show here that PGE_2_ also deactivates these same processes in myofibroblasts (Figures 1, 3, 4, and 7). By contrast, the potent fibroblast mitogen FGF2 (34) stimulates proliferation in myofibroblasts (Figure 4B and Figure 7, A and B). These comparative data suggest that, while FGF2 dedifferentiates lung myofibroblasts primarily by promoting their proliferation, PGE_2_ accomplishes this via a much more global form of cellular deactivation. A major and functionally important consequence of these divergent mechanisms of dedifferentiation is that transitional cells resulting from PGE_2_ exposure are more, while those resulting from FGF2 exposure are less, susceptible to apoptosis (Figure 7C).

It is likely that in order to undergo cell division, dedifferentiating myofibroblasts must restructure their cytoskeletal network and matrix interactions. Indeed, FGF2 downregulated αSMA, stress fibers, and collagen I in established myofibroblasts via activation of the MEK/ERK pathway (Figure 1, B, C, and E), which has also been previously shown to mediate proliferation in myofibroblasts (33). Morphologically, FGF2-treated myofibroblasts elongate and lose their stellate shape, resembling native fibroblasts (Figure 6A). Overexpression of FGF2 has similarly been reported to inhibit myofibroblast differentiation in vitro and pulmonary fibrosis in vivo (14). In contrast to the mechanisms of action of FGF2, PGE_2_ dedifferentiates myofibroblasts (Figure 1D) while concomitantly inhibiting their proliferation (Figure 7B), effects likely to be mediated through cAMP/Epac (35), rather than ERK (Supplemental Figure 1C). In addition to our current findings and our previously published microarray data (12) demonstrating that PGE_2_ remodels the global transcriptome of established myofibroblasts, the importance of cAMP signaling in this regard is further validated by recent parallel findings with a PGI_2_ analog acting via its G_s_-coupled receptor (63).

In addition to modulating mRNA expression, PGE_2_ and FGF2 differentially regulated multiple lncRNAs and miRNAs in lung myofibroblasts (Figure 5). Several ncRNAs modulated in our study have been reported to influence myofibroblast function and fibrotic outcomes. Importantly, many antifibrotic miRNAs that were not significantly modulated by either treatment do, in fact, interact with lncRNAs modulated by PGE_2_ and/or FGF2 (Table 2). The ncRNAs encompass a large family of pleiotropic posttranscriptional regulators that control the expression of gene programs through complex lncRNA-miRNA-mRNA interactions (70, 71). We, therefore, speculate that lncRNAs and miRNAs may be important for the effects of both PGE_2_ and FGF2 in myofibroblasts. Their coordinated actions remain to be more comprehensively defined, and these data provide a blueprint for doing so in future studies.

Since myofibroblast dedifferentiation elicited by PGE_2_ and FGF2 — expected constituents of an injured/fibrotic lung (72, 73) — proceeded via distinct signaling mechanisms and gene expression programs, we assessed their individual and combined effects on *ACTA2* kinetics, myofibroblast morphology, dedifferentiation, proliferation, and apoptosis sensitivity. Indeed, myofibroblasts treated with both mediators displayed an additive reduction in αSMA mRNA, protein, and stress fibers (Figure 6, B and C), while also exhibiting a morphology distinct from that of either treatment alone (Figure 6A). The effects of combined versus individual treatment on specific fibrosis-associated genes ranged from additive inhibition (*ACTA2*) to no additive effect (*NOX4*) to a dominant effect of PGE_2_ (*CTGF* and *VASP*) (Figure 6D). Moreover, PGE_2_ functioned to negate the proliferative and antiapoptotic effects of FGF2 (Figure 7).

The inherently reductionist nature of our in vitro studies limits the conclusions that can be drawn. In an effort to more broadly contextualize such findings, we compared our data to selected transcriptomic differences in lung fibroblasts obtained during an in vivo model of fibrosis resolution. We acknowledge that the in vivo and in vitro models differed with respect to the species utilized and the timing of analysis. Additionally, the in vivo data lack a time point corresponding to peak fibrosis in the young mice, and instead, we compared the data from mice undergoing fibrosis resolution with those from mice with nonresolving fibrosis. Despite these limitations, substantial parallels in the gene signatures of the mesenchymal cells from the 2 models were apparent and intriguing. Fibroblasts from mice undergoing the early stages of fibrosis resolution exhibited an antiproliferative, proapoptotic, and antiadhesion gene signature, which resembled that seen in PGE_2_-treated and PGE_2_ + FGF2–treated myofibroblasts (Figure 4, A and B, and Figure 7A). It is understood that mesenchymal cell responses to PGE_2_ and/or FGF2 in culture and during fibrosis resolution in vivo may involve the differential behaviors of phenotypically distinct fibroblast subpopulations (74, 75). Moreover, the exclusive use of bulk RNA-seq analysis in both models represents an additional limitation of our study, and single cell approaches — as well as flow sorting of isolated fibroblast subpopulations (76) — will be necessary in the future to assess the cellular heterogeneity of mesenchymal responses during dedifferentiation and fibrosis resolution. Additionally, we acknowledge the importance of confirming these findings in human IPF cells.

Although our study was primarily undertaken to gain a better understanding of the process of myofibroblast dedifferentiation, its potential therapeutic ramifications for fibrotic diseases cannot be ignored. Indeed, inhibition of the PGE_2_-degrading enzyme 15-PGDH has recently been shown to attenuate lung fibrosis in vivo and to reduce collagen levels in lung slices from IPF patients (77). Our results therefore suggest that, in principal, activation of the cAMP/PKA pathway and MEK/ERK have the potential to lead to fibrosis resolution. Notably, this raises the possibility that, by blocking FGFR signaling, the antifibrotic drug nintedanib might promote and maintain myofibroblasts, which in turn may limit its therapeutic efficacy. Increasing cAMP can be achieved in a variety of ways pharmacologically, including treatment with the FDA-approved phosphodiesterase inhibitor roflumilast. Pharmacologic strategies to activate ERK are currently under investigation for use as therapeutics in cancer (78, 79). Combined activation of these pathways represents an attractive therapeutic strategy due to their additive effects on myofibroblast dedifferentiation with restoration of apoptosis sensitivity. The potential of these approaches requires further investigation.

In this study, we have compared — for the first time to our knowledge — the transcriptomic, morphologic, and functional changes in PGE_2_- and FGF2-dedifferentiated lung myofibroblasts. Although PGE_2_- and FGF2-treated myofibroblasts differed in their abilities to proliferate and undergo apoptosis, combined treatment resulted in an antiproliferative/proapoptotic phenotype and an additive effect on dedifferentiation. The cell cycle, apoptosis, and focal adhesion gene signatures exhibited by cells from mice undergoing fibrosis resolution more closely resembled those elicited by myofibroblast treatment with PGE_2_ than with FGF2, but they most closely paralleled the effects of combined PGE_2_ + FGF2 treatment in vitro. Mechanistically, PGE_2_ induced dedifferentiation via cAMP-mediated deactivation of myofibroblasts, whereas FGF2 utilized MEK/ERK to prompt dedifferentiation through myofibroblast proliferation. As neither of the current therapies for IPF exploit cAMP signaling, and as nintedanib antagonizes ERK, our results highlight additional pathways and gene programs that hold promise and warrant further investigation in the development of novel therapeutics for this and other fibrotic diseases.

## Methods

### Cell culture.

CCL210 normal adult human lung fibroblasts were obtained from the American Type Culture Collection. Cell culture was performed in low glucose DMEM (Invitrogen) supplemented with 10% FBS (Hyclone), 100 units/mL penicillin, and 100 μg/mL streptomycin (both from Invitrogen). Cells were then serum starved in FBS-free DMEM overnight, and differentiation to myofibroblasts was induced by treatment with TGF-β for 48 hours. TGF-β–elicited myofibroblasts were then treated for specified time points as described followed by harvesting (10).

### Reagents.

Unless otherwise specified, pharmacologic agents were reconstituted in DMSO as stock solutions and stored at –80°C with working concentrations indicated in parentheses. Recombinant human TGF-β (2 ng/mL) and FGF2 (50 ng/mL) were purchased from R&D and stored in filter-sterilized 1% BSA at –20°C. Inhibitors of the MAPKs MEK/ERK (PD98059, 20 μM; U0126, 20 μM) and p38 (SB203580, 20 mM) were purchased from Cayman Chemicals and stored at –20°C. PGE_2_ (1 μM) purged with nitrogen gas, the E-prostanoid 2 agonist butaprost (500 nM), the direct adenylyl cyclase activator forskolin (500 nM), the JNK inhibitor SP600125 (20 mM), and the AKT-1, -2, -3 inhibitor Triciribine (20 mM) were purchased from Cayman Chemicals. The PKA-selective cAMP agonist 6-BNZ cAMP (2 mM) and the Epac-selective agonist 8-pCPT cAMP (2 mM) were purchased from BioLog and reconstituted in sterile water. Fast and Power SYBR Green Master Mix and StepOne real-time PCR system were procured from Applied Biosystems. An αSMA primary antibody conjugated to a FITC-conjugated secondary antibody (F3777, Sigma-Aldrich), polyclonal annexin V antibody (PA5-57231, Thermo Fisher Scientific), PE-conjugated secondary antibody (P2771MP, Thermo Fisher Scientific), and the dyes PKH-26 (Sigma-Aldrich) and Prolong Gold antifade reagent with DAPI (Invitrogen) were used for immunofluorescence microscopy. Apoptosis was induced with anti-Fas activating antibody (CH11, EMD Biosciences).

### qPCR.

qPCR analysis of transcript expression was performed by extracting total cellular RNA using an RNeasy kit (Qiagen). cDNA was prepared using the High Capacity cDNA Reverse Transcription Kit (Applied Biosystems), amplified with Fast SYBR Green Master Mix, and analyzed on a StepOne real time PCR system (Applied Biosystems). Fold changes were normalized to the expression levels of the housekeeping gene GAPDH. Primer pair sequences used for qPCR are listed in Supplemental Table 6.

### Western blot.

For Western blot analysis, cells were lysed in RIPA buffer supplemented with protease inhibitors (Roche Diagnostics) and a phosphatase inhibitor cocktail (EMD Biosciences). Proteins were separated by SDS-PAGE and transferred to a nitrocellulose membrane. Membranes were subsequently blocked with 5% nonfat dry milk or 5% BSA and probed with a mouse antibody specific to αSMA at 1:2000 (ab7817, Abcam) or the rabbit antibodies targeting caspase-3 (CASP3, 9662S), PARP (catalog 9532S), and GAPDH HRP conjugate (catalog 8884S) at 1:1000 (Cell Signaling Technologies). All experiments were performed in triplicate, and the results are presented as mean ± SEM.

### Immunofluorescence microscopy.

CCL210 fibroblasts were seeded and cultured in single-chamber slides (Nunc) followed by overnight serum starvation. Myofibroblasts were generated by incubation with TGF-β for 48 hours and were then treated with PGE_2_ and/or FGF2 for an additional 5 days. Chamber slides were then washed twice with chilled PBS, fixed with freshly prepared 4% formaldehyde for 20 minutes, washed with PBS, and quenched with 100 mM glycine for 15 minutes. Blocking and permeabilization were achieved by incubating the slides for 1 hour in PBS containing 10% FBS and 0.1% Triton X-100 (Sigma-Aldrich). Cells were then incubated with either anti–αSMA-FITC (1:500) overnight at 4°C, anti-annexin V (1 μg/mL) at 37°C for 1 hour followed by anti–rabbit PE–conjugated secondary antibody overnight at 4°C, or the membrane dye PKH26 (2 μM) at room temperature for 3 minutes. Mounting medium containing DAPI was then used to stain the nuclei. Slides were examined using a Leica DC 500-fluorescence microscope equipped with a digital camera.

### Proliferation and apoptosis assays.

Lung myofibroblast proliferation studies were performed using the CyQUANT NF Cell Proliferation Assay Kit (Thermo Fisher Scientific). At the specified harvesting time, cells were detached with 0.25% trypsin and resuspended in HBSS containing CyQUANT NF dye reagent. Cell lysates were then transferred to a 96-well plate and incubated at 37°C for 45 minutes. Cell proliferation was measured by determining fluorescence in a microplate reader with excitation of 485 nm and emission of 530 nm. Lung myofibroblast apoptosis was evaluated following treatment with anti-Fas antibody at 100 ng/mL. Total and cleaved (active) forms of CASP3 and PARP were analyzed by Western blot. All experiments were performed in triplicate, and the results are presented as mean ± SEM.

### RNA-seq.

For RNA-seq performed on in vitro specimens, CCL210 human adult lung fibroblasts were treated with PGE_2_ or FGF2, or they were left untreated. Cells were harvested with Trizol followed by RNA extraction using the miRNeasy Mini kit (Qiagen), according to the manufacturer’s protocol, and submitted for library prep and sequencing. For in vivo RNA-seq studies, fibroblasts were isolated from female FVB Col1α1-GFP transgenic mice — obtained from Derek Radisky (Mayo Clinic) — 30 days following bleomycin-challenge. Both young (2 months) and aged (18 months) mice were used in the study (80). Mouse lungs were digested and Col1α1-GFP^+^ fibroblasts were isolated by FACS. Total RNA was extracted using the RNeasy Micro Kit (Qiagen) according to manufacturer’s protocol and submitted for library prep and sequencing. A detailed description of RNA library preparation, sequencing protocols, and analysis of RNA isolated from CCL210 and mouse fibroblasts is available in the supplement. Our RNA-Seq data were deposited in the NCBI GEO database (accession no. GSE163832).

### Statistics.

Statistical analysis was performed using GraphPad Prism software version 8.1.1. Experimental data are presented as means and were analyzed for statistical significance by 1-way ANOVA with the Tukey’s multiple comparisons test, 2-way ANOVA (Figure 6B), or paired 2-tailed *t* test, as appropriate. *P* < 0.05 was considered significant. Data are presented as ± SEM. For RNA-seq data, DESeq2 was used with default parameters to identify differentially expressed transcripts with cutoffs of Benjamini-Hochberg FDR-adjusted *P* < 0.05 and fold-change values (excluding small RNAs) of less than –1.5 or greater than 1.5 (81). We elected to omit fold-change cutoffs when analyzing small RNAs to capture all statistically significant changes that may have biologic relevance. Functional analysis of all RNAs (except small RNAs), including candidate pathways activated or inhibited in comparisons and GO-term enrichments (http://www.geneontology.org/), was performed using iPathway Guide (http://www.advaitabio.com) (82). See supplemental methods for detailed quality control and sequence alignment methodology.

### Study approval.

All animal experiments were carried out in accordance with the Mayo Clinic IACUC and conforming to the Animal Research: Reporting of In Vivo Experiments (ARRIVE) guidelines.

## Author contributions

SMF, LRP, and MPG designed the in vitro experiments. TXP and GL designed the in vivo experiments. Experiments were performed by SMF, LRP, and TXP. Data were analyzed by SMF and DK. The manuscript was written by SMF and MPG.

## Supplementary Material

Supplemental data

## Figures and Tables

**Table 1:**
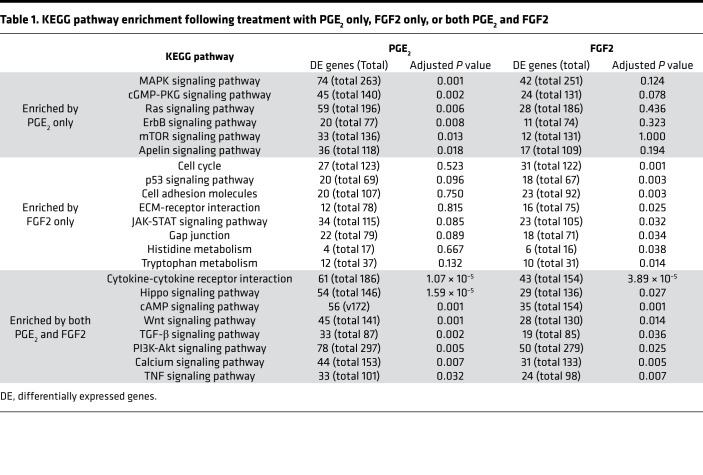
KEGG pathway enrichment following treatment with PGE_2_ only, FGF2 only, or both PGE_2_ and FGF2

**Table 2:**
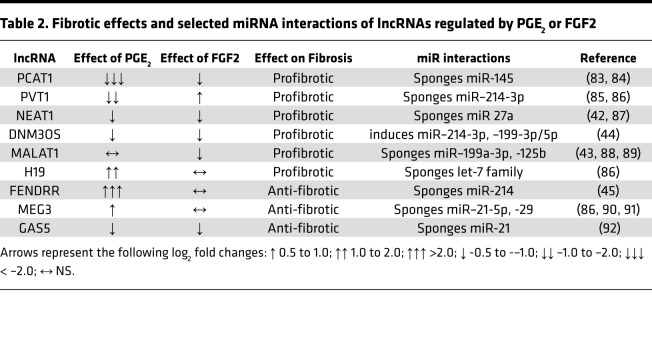
Fibrotic effects and selected miRNA interactions of lncRNAs regulated by PGE_2_ or FGF2

## Version 1 02/09/2021

In-Press Preview

## Version 2 03/22/2021

Electronic publication
