# Supplementary material for: Myofibroblast dedifferentiation proceeds via distinct transcriptomic and phenotypic transitions
Source: JCI Insight. 2021 Mar 22;6(6):e144799. doi: 10.1172/jci.insight.144799 (PMC8026183; doi:10.1172/jci.insight.144799)
Supplement: Supplemental data [file jciinsight-6-144799-s100.pdf]

Myofibroblast de-differentiation proceeds via distinct transcriptomic and phenotypic transitions

Sean M. Fortier, Loka R. Penke, Dana King, Tho X. Pham, Giovanni Ligresti, Marc Peters-Golden

Supplemental Methods, Figures, and Tables

Supplemental Methods

Total and Small RNA Isolation – CCL210 Fibroblasts

For RNA extraction, each experimental condition was performed in triplicate and cells were harvested in Trizol (Qiagen, Germantown, MD). RNA extraction was performed using the Qiagen miRNeasy Mini kit per the manufacturer's instructions to extract all RNA moieties >18 nucleotides. Genomic DNA was digested on-column per the manufacturer's instructions using the RNase-Free DNase Set (New England Biolabs, Ipswitch, MA). RNA concentration was measured using NanoDrop and RNA integrity was measured using BioAnalyzer (Agilent, Santa Clara, CA) and submitted for library preparation and sequencing.

Total RNA Library Preparation – CCL210 Fibroblasts

RNA was assessed for quality using the TapeStation (Agilent, Santa Clara, CA). Samples were prepared using the NEBNext Ultra II Directional RNA Library Prep Kit for Illumina (catalog number E7760L) Ribo depletion Module NEBNext rRNA Human/Mouse/Rat (catalog number E6310X) and NEBNext Multiplex Oligos for Illumina Unique dual (catalog number E6440L) (NEB, Ipswitch, MA) where 370 ng of total RNA was ribosomal depleted using rRNA Depletion module. The rRNA-

depleted RNA was then fragmented prior to cDNA generation. NEBNext adapters were added via ligation and products were purified and enrichment with PCR (11 cycles) to create the final cDNA library. Final libraries were checked for quality and quantity by TapeStation (Agilent) and qPCR using Kapa's library quantification kit for Illumina Sequencing platforms (catalog # KK4835) (Kapa Biosystems, Wilmington MA). The samples were pooled and sequenced on the Illumina NovaSeq S4 Paired-end 150 bp, according to manufacturer's recommended protocols.

Small RNA Library Preparation – CCL210 Fibroblasts

RNA was assessed for quality using the TapeStation (Agilent) using manufacturer's recommended protocols. Samples were prepared using the NEBNext Multiplex Small RNA Library Prep Set for Illumina (catalog number E7300L). Adapters were ligated to 500 ng of total RNA which then goes through 1st Strand Synthesis and 12 cycles PCR amplification. The products are purified and size selected by Pippin Prep according to NEB protocol recommendations. Final libraries were checked for quality and quantity by TapeStation (Agilent) and qPCR using Kapa's library quantification kit for Illumina Sequencing platforms (catalog # KK4835) (Kapa Biosystems, Wilmington MA). The samples were pooled and sequenced on the

Illumina NovaSeq S4 Paired-end 150 bp, according to manufacturer's recommended protocols.

Differential Expression Analysis, Total RNA – CCL210 Fibroblasts

Analysis of all CCL210 Fibroblast RNA-seq data was performed by the University of Michigan Bioinformatics Core. Raw reads were downloaded from the Advanced Genomics' Core storage and adapter sequences were trimmed using Cutadapt (v2.3). FastQC (1) (v0.11.8) was used to ensure data quality. Reads were mapped to the reference genome hg19 (UCSC) using STAR (2) (v2.6.1b) and assigned count estimates to genes with RSEM (3) (v1.3.1). For "totalRNA" results, gene reference included both mRNAs and some lncRNAs. For "lncRNA_specific" results, gene reference was limited to GENCODE transcripts annotated as long noncoding RNAs (https://www.gencodegenes.org/human/release_19.html). Alignment options followed ENCODE standards for RNA-seq (https://github.com/alexdobin/STAR/blob/master/doc/STARmanual.pdf). FastQC was used in an additional post-alignment step to ensure that only high-quality data were used.

For differential expression analysis, data were pre-filtered to remove genes with 0 counts in all samples. Differential gene expression analysis was performed using

DESeq2 (4), using a negative binomial generalized linear model with cutoffs of linear fold change > 1.5 or < -1.5, Benjamini-Hochberg FDR (Padj) < 0.05. Plots were generated using variations of DESeq2 plotting functions and other packages with R version 3.3.3. For 'totalRNA' results, genes were annotated with NCBI Entrez GeneIDs and text descriptions. For 'totalRNA' results, functional analysis, including KEGG pathway and GO-term enrichments (5), was performed using iPathway Guide (Advaita) (6, 7).

Differential Expression Analysis, Small RNA – CCL210 Fibroblasts

Data was analyzed using the CAP-miRSeq pipeline from the Mayo clinic (8), using human reference genome version hg19 (UCSC), and MirBase version 2 (9). Briefly, FastQC was used to ensure data quality. Reads were trimmed using Cutadapt. The miRDeep2 mapper and miRDeep2 module (10) are the core components for known and novel miRNA detection. Differential miRNA analysis was performed using edgeR (11). TargetScan 7.2 (12) (http://www.targetscan.org/) was used for predicted targets, and MiR-TarBase 8.0 (13) (http://mirtarbase.cuhk.edu.cn/) was used for experimentally validated targets.

RNA Isolation – Mouse Lung Fibroblasts

Young (2 mo) and aged (18 mo) Col1α1-GFP mice were treated with bleomycin by intratracheal administration to induce fibrosis as previously described (14); mice receiving PBS instead of bleomycin were used as controls. Thirty days after bleomycin treatment, at the early stages of fibrosis resolution (14), mice were anaesthetized with 100 mg/kg ketamine and 10 mg/kg xylazine. The lungs were perfused with cold PBS, harvested, minced, and subsequently digested in cold DMEM media containing 0.2 mg/mL Liberase DL and 100 U/mL DNase I (Roche, Basel, Switerzerland) for 35 minutes. Red blood cells were removed from the cell suspension with red blood cell lysis buffer (Biolegend, San Diego, CA). The single cell suspension was then incubated with DAPI (1:1000), anti-CD45: PerCp-Cy5.5 (1:200), antiCD31:PE (1:200), and anti-CD326:APC (1:200) antibodies (Biolegend) for 30 min on ice. FACS sorting was conducted using a BD FACS Aria II (BD Biosciences, San Jose, CA). DAPI-/CD45-/CD326-/CD31-/GFP+ selection was used to sort Col1α1-GFP+ mouse lung cells. The cells were sorted straight into lysis buffer from the RNeasy Micro Kit (Qiagen) and total RNA was isolated according to manufacturer's protocol. Total RNA isolated from sorted DAPI-/CD45-/CD326-/CD31-/GFP+ lung cells were used for library prep and sequencing.

RNA Library Preparation – Mouse Lung Fibroblasts

RNA quality was determined using the Fragment Analyzer from AATI (AATI, Ankeny, IA) and only samples with RQN values ≥ 6 were used. Sequencing libraries were prepared using 200 ng of total RNA with the TruSeq RNA Sample Prep Kit v2 (Illumina) and poly-A mRNA enrichment according to the manufacturer's protocol. Pair-end reads of 2x100 bp were sequenced on an Illumina HiSeq 4000 using the HiSeq 3000/4000 sequencing kit, yielding 33-40 million fragment reads per sample.

Differential Expression Analysis – Mouse Lung Fibroblasts

Analysis of RNAseq data from mouse Col1α1-GFP+ lung fibroblasts, Mayo Clinic's MAPR-Seq software was used to process the raw paired end reads from the RNA sequencing experiments. The raw gene count expression values from MAPR-Seq were then processed by the R package, edgeR, to evaluate differential expression. Genes with an average raw gene count less than 25 in the samples were excluded from the differential expression analysis. Differentially expressed genes between the young and aged groups were identified using Smyth's moderated t test and Benjamini-Hochberg procedure for adjusted P value (FDR). Genes with a false

discovery rate (FDR) below 0.05, and absolute log2 fold change greater than 1 were defined as being differentially expressed.

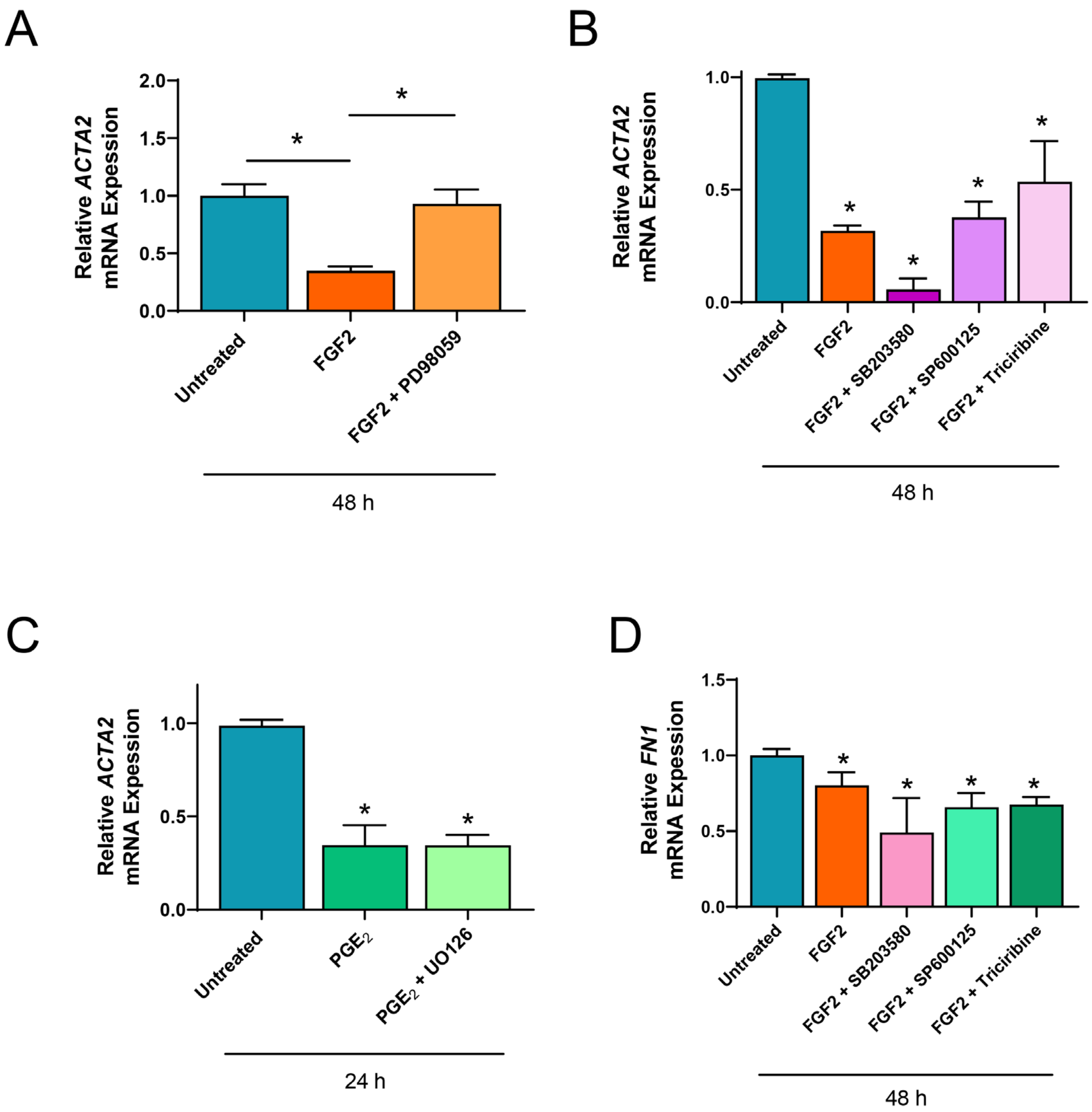

Supplemental Figure 1: (**A** and **C**) FGF2 reduces *ACTA2* in myofibroblasts via MEK/ERK whereas PGE_2 effects on ACTA2 are unaffected by MEK/ERK. (**A**) The MEK/ERK inhibitor PD98059 and (C) UO126 were used at working concentrations of 20 µM. (**B** and **D**) *ACTA2* and *FN1* reduction by FGF2 does not depend on AKT, JNK, or p38. The AKT inhibitor Triciribine, JNK inhibitor SP600125, and p38

inhibitor SB203580 were used at working concentrations of 20 μM. Bars represent mean ± SEM; black circles in represent replicated samples from three experiments. Lines indicate conditions being compared. Isolated asterisk indicates statistical significance compared with untreated myofibroblast. *P < 0.05.

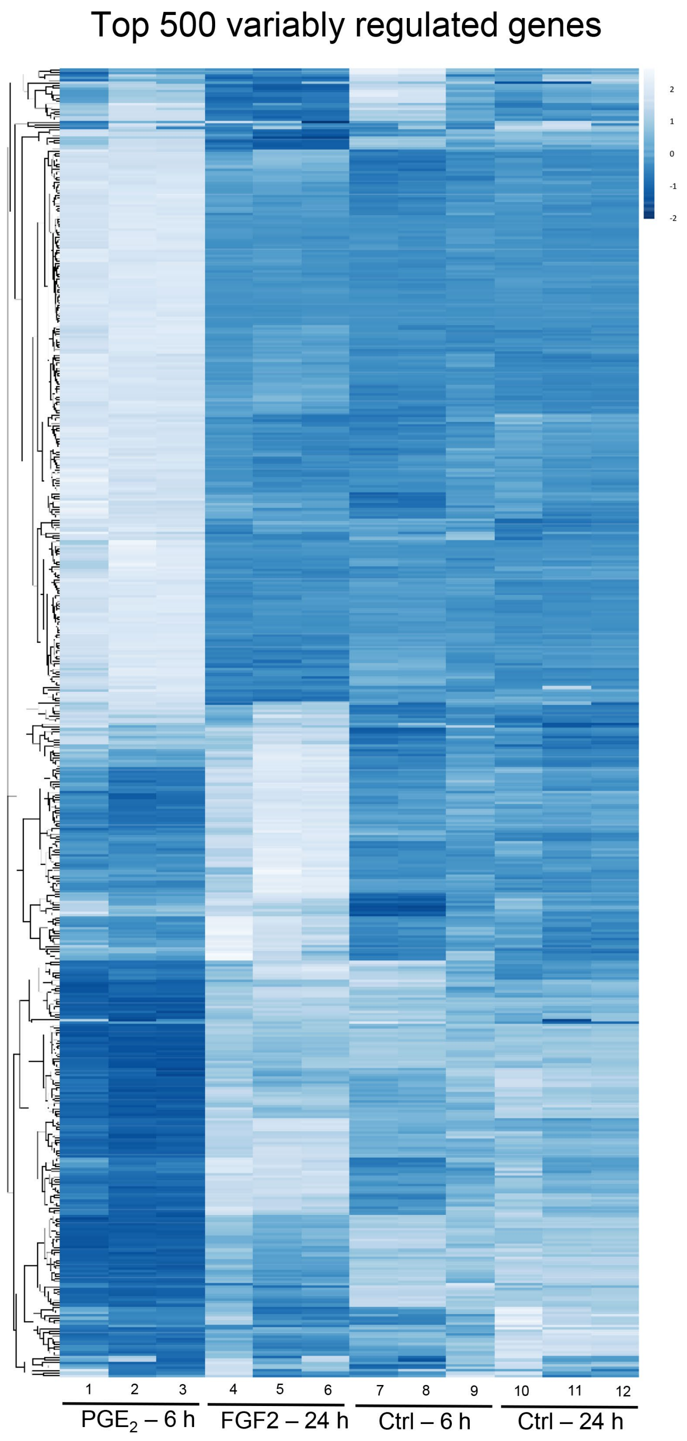

Supplemental Figure 2: Heatmap display of four experimental conditions upon which RNA-seq was performed in triplicate.

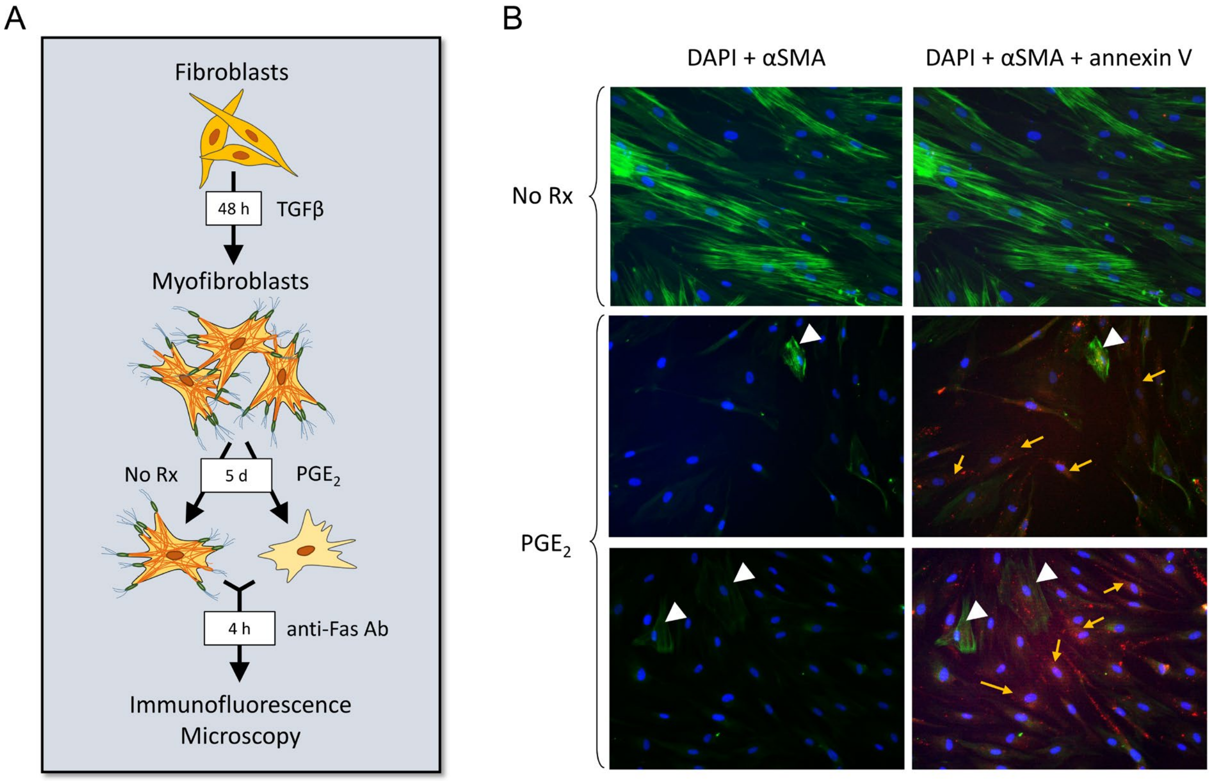

Supplemental Figure 3: PGE_2 sensitizes myofibroblasts to apoptosis following de-differentiation. (**A**) Experimental scheme depicting myofibroblast differentiation by TGFβ followed by a 5 d treatment with or without PGE_2; de-differentiated myofibroblasts and myofibroblasts (untreated controls) were then treated with anti-Fas Ab for 4 h to induce apoptosis and were assessed for αSMA stress fiber and annexin V expression by immunofluorescence microscopy. (**B**) αSMA stress fibers identified by immunofluorescence microscopy (left column) and annexin V staining merged with αSMA (right column) in untreated and PGE_2-treated myofibroblasts. The two bottom rows represent different microscopic fields of PGE_2-treated myofibroblasts. White arrowheads represent PGE_2-treated

myofibroblasts with persistent stress fiber expression. Yellow arrows represent selected examples of annexin V stained cells. Stress fibers were stained using anti-αSMA antibody and FITC-conjugated secondary antibody and annexin V was stained using a polyclonal antibody followed by PE-conjugated secondary antibody. Nuclei are stained with DAPI. Images are 20x magnification. No Rx = untreated controls.

Rank	PGE_2						FGF2					
	Upregulated			Downregulated			Upregulated			Downregulated		
	Gene	Log2	Adjusted p-value	Gene	Log2FC	Adjusted p-value	Gene	Log2FC	Adjusted p-value	Gene	Log2FC	Adjusted p-value
1	AREG	8.133	<1.0E-300	DEPP1	-4.598	6.7E-194	EDN1	3.095	7.2E-66	IGF1	-2.354	1.8E-13
2	IL33	7.697	8.4E-198	ANKRD1	-4.116	1.6E-48	KPRP	3.020	2.5E-13	TM4SF20	-2.188	6.0E-23
3	NR4A2	6.391	2.7E-268	B3GALT2	-3.894	3.4E-261	PKMYT1	2.814	3.2E-15	GUCY1B3	-2.050	8.0E-19
4	SLCO4A1	5.931	8.4E-68	GCNT4	-3.548	2.4E-64	FAM111B	2.798	2.2E-14	ARHGAP28	-2.018	9.4E-12
5	NFATC2	5.707	1.1E-170	NAV3	-3.547	1.3E-148	RRM2	2.777	3.1E-14	SLC40A1	-1.987	4.1E-07
6	STC1	5.691	2.9E-170	EGR2	-3.489	1.6E-32	ASF1B	2.692	7.5E-18	FAM179A	-1.986	6.7E-08
7	MLC1	5.646	2.2E-97	RTKN2	-3.469	2.3E-51	GMCSF	2.691	8.3E-11	LCNL1	-1.972	6.0E-22
8	TNFRSF18	5.488	7.3E-70	SERTAD4	-3.370	1.6E-87	MCM10	2.597	3.7E-12	DEPP1	-1.938	2.7E-41
9	NR4A3	5.374	1.1E-105	FAM84B	-3.356	2.0E-37	IL8	2.582	2.9E-12	ICOSLG	-1.907	3.4E-12
10	LYPD3	5.230	2.8E-149	KLF5	-3.281	8.9E-106	CLSPN	2.573	2.3E-24	DIRAS2	-1.882	3.4E-09
11	SH2D2A	5.164	2.0E-45	TRIB3	-3.273	7.3E-250	NT5E	2.474	3.3E-35	GUCY1A3	-1.860	3.2E-06
12	SST	5.115	3.4E-43	VDR	-3.246	1.2E-178	ZNF367	2.448	4.0E-12	PDE1A	-1.848	2.4E-06
13	WNT1	5.075	2.1E-43	PKP2	-3.199	1.7E-40	SLC14A1	2.399	6.2E-14	MMP28	-1.825	2.3E-13
14	FAM167A	5.066	9.3E-157	SGK223	-3.179	4.1E-121	BIRC3	2.385	9.7E-32	ATP1A2	-1.807	4.6E-07
15	MUC13	4.956	3.6E-41	NLRP10	-3.109	2.6E-34	PLAT	2.381	9.8E-36	MAOB	-1.736	2.3E-11
16	CHGB	4.940	3.8E-41	DRD1	-2.794	5.0E-15	MPP4	2.380	9.2E-14	KLHL38	-1.718	1.5E-06
17	SPOCK2	4.936	3.2E-79	GADD45A	-2.779	8.2E-74	CNIH3	2.370	2.6E-19	BCL2L11	-1.708	9.0E-24
18	IQSEC3	4.846	7.9E-282	SYNPO2L	-2.705	1.5E-28	KIFC1	2.338	6.9E-18	CRISPLD2	-1.695	3.2E-09
19	MEDAG	4.844	1.0E-206	GPR39	-2.700	2.0E-57	AURKB	2.335	1.4E-08	TNFAIP8	-1.694	7.7E-27
20	TM4SF1	4.836	5.6E-85	ARNT2	-2.686	2.9E-51	DTL	2.328	1.6E-12	CXCR7	-1.681	2.2E-05
21	TNS4	4.671	5.5E-38	KCTD16	-2.674	1.1E-50	TNFRSF6B	2.312	1.8E-13	PTGER3	-1.681	3.6E-05
22	PNPLA5	4.533	4.4E-43	CDC42EP3	-2.629	<1.0E-300	COL13A1	2.279	4.5E-18	NPR3	-1.677	1.0E-08
23	CHL1	4.526	6.2E-74	JPH2	-2.625	2.6E-24	KRTAP1-5	2.276	3.2E-08	FBXL22	-1.676	1.2E-06
24	ZMAT4	4.483	3.6E-33	IFIT2	-2.606	1.4E-20	FOSL1	2.273	2.9E-38	MAF	-1.672	1.3E-09
25	SMOC1	4.471	4.6E-267	GPR63	-2.585	1.0E-17	MYBL2	2.265	1.8E-08	SAMD11	-1.643	1.1E-06

Supplemental Table 1: Top 25 modulated myofibroblast mRNAs following treatment with PGE_2 or FGF2.

	KEGG Pathway	PGE_2		FGF2	
		DE genes (Total)	Adjusted p-value	DE genes (Total)	Adjusted p-value
Enriched by PGE2 Only	Proteoglycans in cancer	55 (180)	5.24E-04	29 (171)	0.08
	Morphine addiction	26 (70)	5.24E-04	9 (62)	0.716
	Melanogenesis	29 (90)	5.24E-04	15 (82)	0.283
	Signaling pathways regulating pluripotency of stem cells	41 (121)	8.08E-04	17 (114)	0.317
	MAPK signaling pathway	74 (263)	0.001	42 (251)	0.124
	cGMP-PKG signaling pathway	45 (140)	0.002	24 (131)	0.078
	Colorectal cancer	25 (83)	0.003	16 (82)	0.212
	Ras signaling pathway	59 (196)	0.006	28 (186)	0.436
	GABAergic synapse	19 (64)	0.006	6 (54)	0.955
	Hepatocellular carcinoma	37 (153)	0.007	25 (147)	0.209
	Gastric acid secretion	17 (60)	0.008	10 (50)	0.189
	ErbB signaling pathway	20 (77)	0.008	11 (74)	0.323
	mTOR signaling pathway	33 (136)	0.013	12 (131)	1.000
	Estrogen signaling pathway	33 (109)	0.013	16 (104)	0.365
	Apelin signaling pathway	36 (118)	0.018	17 (109)	0.194
	Central carbon metabolism in cancer	23 (61)	0.026	5 (61)	0.436
	Cholinergic synapse	27 (91)	0.028	11 (81)	0.269
	Hippo signaling pathway - mupltiple species	10 (27)	0.033	5 (25)	0.189
	Insulin resistance	26 (92)	0.033	15 (88)	0.365
	Circadian entrainment	25 (85)	0.033	11 (73)	0.446
	Serotonergic synapse	23 (78)	0.034	13 (68)	0.295
	Relaxin signaling pathway	26 (114)	0.036	15 (108)	0.639
	Thyroid hormone signaling pathway	29 (104)	0.042	14 (102)	0.520
	Ovarian steroidogenesis	12 (36)	0.044	7 (32)	0.436
	Mineal absorption	11 (39)	0.044	6 (33)	0.322
	EGFR tyrosine kinase inhibitor resistance	25 (73)	0.044	10 (72)	0.666
	Melanoma	23 (66)	0.045	10 (61)	0.593
	Prolactin signaling pathway	20 (63)	0.045	8 (62)	0.545
	Osteoclast differentiation	29 (98)	0.045	18 (90)	0.103
Enriched by FGF2 Only	Hypertrophic cardiomyopathy	15 (72)	0.621	21 (68)	6.85E-04
	Systemic lupus erythematous	13 (103)	0.713	26 (90)	0.001
	Dilated cardomyopathy	14 (75)	0.590	22 (70)	0.001
	Renin secretion	14 (55)	0.622	15 (52)	0.001
	Cell cycle	27 (123)	0.523	31 (122)	0.001
	p53 signaling pathway	20 (69)	0.096	18 (67)	0.003
	Cell adhesion molecules	20 (107)	0.750	23 (92)	0.003
	Human T-cell leukemia virus 1 infection	45 (192)	0.273	39 (181)	0.004
	microRNAs in cancer	37 (140)	0.070	30 (138)	0.004
	Inflammatory bowel disease	13 (38)	0.212	9 (30)	0.004
	Inflammatory mediator regulation of TRP channels	24 (87)	0.109	20 (80)	0.005
	Alcoholism	35 (161)	0.643	33 (155)	0.005
	Rheumatoid arthritis	18 (65)	0.367	16 (55)	0.005
	AGE-RAGE signaling pathway in diabetic complications	23 (96)	0.444	24 (94)	0.008
	Pathogenic *Escherichia coli* infection	12 (51)	0.109	9 (48)	0.010
	Tyrptophan metabolism	12 (37)	0.132	10 (31)	0.014
	ECM-receptor interaction	12 (78)	0.815	16 (75)	0.025
	Complement and coagulation cascades	13 (59)	0.760	14 (48)	0.025
	Interstinal immune network for IgA production	4 (25)	1.000	8 (20)	0.027
	JAK-STAT signaling pathway	34 (115)	0.085	23 (105)	0.032
	Th1 and Th2 cell differentiation	16 (69)	0.701	11 (59)	0.032
	Insulin secretion	20 (66)	0.054	14 (57)	0.034
	Gap junction	22 (79)	0.089	18 (71)	0.034
	Bile secretion	10 (47)	0.498	11 (40)	0.034
	Adrenergic signaling pathway	28 (117)	0.389	24 (112)	0.035
	Histidine metabolism	4 (17)	0.666	6 (16)	0.038
	Aldosterone-regulated sodium reabsorption	9 (29)	0.249	4 (25)	0.042
	Leishmaniasis	9 (48)	0.711	10 (40)	0.050
	Mucin type O-glycan biosynthesis	10 (25)	0.062	7 (22)	0.050
Enriched by both PGE_2 and FGF2	Cytokine-cytokine receptor interaction	61 (186)	1.07E-05	43 (154)	3.90E-05
	Breast cancer	49 (131)	1.31E-05	28 (122)	0.004
	Hippo signaling pathway	54 (146)	1.59E-05	29 (136)	0.027
	Basal cell carcinoma	24 (58)	4.12E-04	15 (54)	0.013
	Neuroactive ligand-receptor interaction	21/76	4.13E-04	36 (157)	0.003
	Gastric cancer	42 (129)	5.23E-04	25 (119)	0.027
	cAMP signaling pathway	56 (172)	5.24E-04	35 (154)	0.001
	Wnt signaling pathway	45 (141)	5.45E-04	28 (130)	0.014
	Pathways in cancer	123 (465)	6.05E-04	93 (440)	6.85E-04
	Cushing syndrome	40 (136)	0.001	26 (126)	0.028
	TGFβ signaling pathway	33 (87)	0.002	19 (85)	0.036
	PI3K-Akt signaling pathway	78 (279)	0.005	50 (279)	0.025
	Calcium signaling pathway	44 (153)	0.007	31 (133)	0.005
	TNF signaling pathway	33 (101)	0.032	24 (98)	0.007
	Axon guidance	49 (169)	0.033	38 (164)	0.002
	Hematopoietic cell lineage	18 (54)	0.045	18 (44)	5.15E-05
	Transcriptional misregulation in cancer	43 (159)	0.047	36 (149)	9.45E-04

Supplemental Table 2: KEGG pathways enriched by PGE_2 only, FGF2 only, and by both PGE_2 and FGF2.

Rank	PGE_2						FGF2					
	Upregulated			Downregulated			Upregulated			Downregulated		
	Gene	Log2	Adjusted p-value	Gene	Log2FC	Adjusted p-value	Gene	Log2FC	Adjusted p-value	Gene	Log2FC	Adjusted p-value
1	LINC00602	6.732	3.8E-80	MYHAS	-3.596	2.2E-16	AL365361.1	3.230	5.7E-15	HELLPAR	-2.672	9.5E-12
2	AC018629.1	4.602	2.8E-24	PCAT1	-3.078	1.0E-18	SFTA1P	2.803	2.7E-14	ADAMTS9-AS1	-2.405	1.1E-06
3	AL358334.2	4.391	5.4E-23	LINC01614	-2.953	1.7E-20	LINC02454	2.339	9.5E-11	AL109924.1	-2.083	1.8E-05
4	AC026369.1	4.264	4.4E-52	AC117488.1	-2.819	2.1E-12	LNCOG	2.332	9.5E-53	AC018647.1	-1.943	6.3E-04
5	GPRACR	4.087	4.5E-24	AL035681.1	-2.767	2.8E-44	DIRC3-AS1	2.303	1.5E-23	LINC01914	-1.931	5.6E-05
6	AC004817.3	4.003	7.7E-23	C7orf69	-2.733	1.4E-18	LINC01605	2.294	1.4E-09	ID2-AS1	-1.829	4.6E-05
7	AL050404.1	3.877	2.8E-71	AC087636.1	-2.672	1.3E-11	AC105383.1	2.272	5.2E-11	AC100803.3	-1.799	5.1E-04
8	OVCH1-AS1	3.741	2.0E-17	LINC01936	-2.525	3.9E-19	AC016397.2	2.267	1.1E-08	AC008063.1	-1.656	1.9E-03
9	AL360178.2	3.726	1.2E-18	AC108451.1	-2.482	6.0E-08	HECW2-AS1	2.227	2.5E-15	LINC01936	-1.644	1.1E-10
10	CHMP1B-AS1	3.346	2.4E-66	MANCR	-2.323	1.4E-06	AC024909.1	2.208	3.4E-12	BX842242.1	-1.623	1.2E-02
11	AL390783.1	3.331	4.8E-10	AL035447.1	-2.298	3.7E-08	MANCR	2.107	1.2E-06	AC024597.1	-1.588	1.2E-03
12	LINC00161	3.329	1.4E-10	AC024598.1	-2.201	9.5E-25	AC008966.3	1.994	9.7E-05	AC120049.1	-1.573	1.6E-02
13	AC008149.2	3.296	6.5E-47	AC117453.1	-2.170	1.8E-05	MIR222HG	1.969	2.0E-19	AC009961.1	-1.561	4.9E-03
14	AL138828.1	3.279	4.7E-14	AC093879.1	-2.168	2.1E-06	AC016831.1	1.956	1.6E-21	LINC02593	-1.560	2.5E-08
15	AC129102.1	3.250	2.2E-10	PLCE1-AS1	-2.146	2.8E-07	AC091182.2	1.946	1.5E-03	AC018697.1	-1.557	2.3E-03
16	AC003092.1	3.109	1.1E-09	GATA6-AS1	-2.128	4.7E-10	APCDD1L-DT	1.938	1.3E-14	Z99289.2	-1.493	4.7E-04
17	TEX26-AS1	3.051	2.5E-09	AP000577.2	-2.063	2.4E-11	LINC00973	1.873	6.2E-05	AL354861.3	-1.447	3.4E-03
18	LINC00545	3.032	2.6E-09	AC005280.1	-2.061	7.5E-11	LINC01204	1.856	2.9E-03	AP005717.1	-1.435	8.9E-03
19	AC004585.1	2.973	2.2E-08	PRAL	-2.047	8.1E-65	AL035665.1	1.791	2.5E-04	AC117453.1	-1.395	1.1E-02
20	AC025259.3	2.911	1.6E-16	MIR181A2HG	-1.998	7.5E-06	L34079.3	1.734	3.4E-03	RBMS3-AS3	-1.370	3.2E-03
21	AL353150.1	2.850	4.4E-15	AC007938.2	-1.988	7.3E-11	AC005280.3	1.721	1.7E-06	AC099552.5	-1.369	2.7E-02
22	AC026369.2	2.850	1.8E-07	AC091152.4	-1.971	1.1E-06	AL139147.1	1.701	8.4E-09	RTCA-AS1	-1.363	8.4E-05
23	AL391056.1	2.792	9.9E-09	AC007744.1	-1.965	4.8E-04	RGMB-AS1	1.688	2.7E-06	AL133390.1	-1.356	4.9E-03
24	AC010735.2	2.757	2.2E-41	AC099506.1	-1.963	2.9E-05	AC008522.1	1.665	1.6E-08	LINC02447	-1.353	2.8E-02
25	LINC00707	2.734	2.9E-07	AP001189.1	-1.939	2.3E-05	AC234772.2	1.646	4.0E-04	LINC01750	-1.344	2.3E-03

Supplemental Table 3: Top 25 modulated long non-coding RNAs following treatment with PGE_2 or FGF2

PGE$_2$		
miR	logFC	Adjusted p-value
miR-129-5p	1.145	1.0E-04
miR-543	0.823	0.017
miR-335-3p	-0.843	0.001
**FGF2**		
miR	logFC	Adjusted p-value
miR-29b-5p	0.908	6.0E-06
miR-188-5p	0.782	0.009
miR-1268a	0.742	5.3E-08
miR-1268b	0.716	1.1E-07
miR-29b-3p	0.701	1.4E-11
miR-335-3p	0.600	1.9E-16
miR-487a-3p	0.594	0.002
miR-543	0.574	4.65E-04
miR-4521	0.559	0.024
miR-376a-3p	0.527	0.029
miR-3117-3p	0.487	0.005
let-7a-3p	0.485	0.031
miR-362-5p	0.456	0.042
miR-222-3p	0.433	3.5E-05
miR-152-5p	0.393	0.049
miR-137	0.360	0.011
miR-199b-5p	0.234	0.027
miR-221-3p	0.209	0.049
miR-145-3p	-0.243	0.027
miR-30e-3p	-0.259	0.044
miR-23b-5p	-0.376	0.019
miR-27b-5p	-0.496	1.57E-04
let-7c-3p	-0.640	4.10E-04

Supplemental Table 4: Complete list of differentially regulated microRNAs by PGE$_2$ and FGF2.

Rank	microRNA	baseMean	Relative Abundance
1	miR-21-5p	234276	100
2	miR-100-5p	102129	43.6
3	miR-143-3p	86087	36.7
4	let-7a-5p	60225	25.7
5	miR-26a-5p	43190	18.4
6	let-7f-5p	37191	15.9
7	miR-27b-3p	36799	15.7
8	let-7i-5p	28026	12.0
9	miR-24-3p	22845	9.8
10	miR-10a-5p	22671	9.7
11	miR-125b-5p	20179	8.6
12	miR-27a-3p	19702	8.4
13	let-7g-5p	17543	7.5
14	let-7b-5p	17090	7.3
15	miR-221-3p	16454	7.0
16	miR-199b-3p	13495	5.8
17	miR-22-3p	12427	5.3
18	miR-29a-3p	12014	5.1
19	miR-199a-5p	7929	3.4
20	miR-127-3p	7566	3.2
21	miR-99a-5p	6318	2.7
22	miR-222-3p	6109	2.6
23	miR-125a-5p	6109	2.6
24	miR-23a-3p	5378	2.3
25	miR-151a-3p	5182	2.2

Supplemental Table 5: Top 25 most abundantly expressed microRNAs in CCL210 human lung myofibroblasts.

Gene	Forward sequence (5' to 3')	Reverse sequence (5' to 3')
GAPDH	CAGCCTCAAGATCATCAGCA	ACAGTCTTCTGGGTGGCAGT
ACTA2	ATCACCAACTGGGACGACAT	CATACATGGCTGGGACATTG
COL1A1	CTGCTGGCAAGAGTGGTGAT	GGTGACCCTTTATGCCTCTG
FN1	CACCACAGCCATCTCACATT	CTGGCCCTCGTATACCACAC
CCNB2	AGTTCCAGTTCAACCCACCA	TGTCCTCGATTTTGCAGAGC
CCND1	CGTGGCCTCTAAGATGAAGG	CCACTTGAGCTTGTTCACCA
CKDN1C	CACTCGGGGATTTCGGGAC	CTTGCGCTTGGCGAAGAAAT
FOXM1	GCAGGCTGCACTATCAACAA	TCGAAGGCTCCTCAACCTTA
BIRC5	CCACTGAGAACGAGCCAGAC	GACAGAAAGGAAAGCGCAAC
CASP9	CTAGTTTGCCCACACCCAGT	GCATTAGCGACCCTAAGCAG
MYC	ATTCTCTGCTCTCCTCGACG	GCCTGCCTCTTTTCCACAGA
SERPINE1	TCTGCCCTCACCAACATTCT	CGGTCATTCCCAGGTTCTCT
CTGF	CACAAGGGCCTCTTCTGTGA	GTACTTGCAGCTGCTCTGGA
NOX4	ACGTTGCATGTTTCAGGAGG	CTGGGTAAACTCTGCCGGTT
VASP	GGGAGAAGAACAGCACAACC	AGCTCCTGTTTCACCCTCTG

Supplemental Table 6: Forward and reverse primers used for qPCR

1. Andrews S. FastQC: a quality control tool for high throughput sequence data. http://www.bioinformatics.babraham.ac.uk/projects/fastqc

2. Dobin A, et al. STAR: ultrafast universal RNA-seq aligner. *Bioinformatics*. 2012;29(1):15-21.

3. Li B, Dewey CN. RSEM: accurate transcript quantification from RNA-Seq data with or without a reference genome. *BMC Bioinformatics*. 2011/08/04 2011;12(1):323.

4. Love MI, et al. Moderated estimation of fold change and dispersion for RNA-seq data with DESeq2. *Genome Biol*. 2014;15(12):550.

5. Ashburner M, et al. Gene ontology: tool for the unification of biology. The Gene Ontology Consortium. *Nat Genet*. May 2000;25(1):25-9.

6. Ahsan S, Drăghici S. Identifying Significantly Impacted Pathways and Putative Mechanisms with iPathwayGuide. *Curr Protoc Bioinformatics*. Jun 27 2017;57:7.15.1-7.15.30.

7. Draghici S, et al. A systems biology approach for pathway level analysis. *Genome Res*. Oct 2007;17(10):1537-45.

8. Sun Z, et al. CAP-miRSeq: a comprehensive analysis pipeline for microRNA sequencing data. *BMC Genomics*. 2014/06/03 2014;15(1):423.

9. Kozomara A, et al. miRBase: from microRNA sequences to function. *Nucleic Acids Res*. Jan 8 2019;47(D1):D155-d162.

10. Friedländer MR, et al. miRDeep2 accurately identifies known and hundreds of novel microRNA genes in seven animal clades. *Nucleic Acids Res*. Jan 2012;40(1):37-52.

11. Robinson MD, et al. edgeR: a Bioconductor package for differential expression analysis of digital gene expression data. *Bioinformatics*. Jan 1 2010;26(1):139-40.

12. Agarwal V, et al. Predicting effective microRNA target sites in mammalian mRNAs. *Elife*. Aug 12 2015;4

13. Huang H-Y, et al. miRTarBase 2020: updates to the experimentally validated microRNA-target interaction database. *Nucleic acids research*. 2020/01// 2020;48(D1):D148-D154.

14. Caporarello N, et al. PGC1alpha repression in IPF fibroblasts drives a pathologic metabolic, secretory and fibrogenic state. *Thorax*. Aug 2019;74(8):749-760.
